# Nitrogen‐Doped TiO_2−_ *_x_* (B)/MXene Heterostructures for Expediting Sulfur Redox Kinetics and Suppressing Lithium Dendrites

**DOI:** 10.1002/advs.202406475

**Published:** 2024-07-23

**Authors:** Mengmeng Zhen, Xiaoyu Wang, Qihang Yang, Zihang Zhang, Zhenzhong Hu, Zhenyu Li, Zhongchang Wang

**Affiliations:** ^1^ School of Energy and Environmental Engineering Hebei University of Technology Tianjin 300071 P. R. China; ^2^ The Center of Functional Materials for Working Fluids of Oil and Gas Field Sichuan Engineering Technology Research Center of Basalt Fiber Composites Development and Application School of New Energy and Materials Southwest Petroleum University Chengdu 610500 China; ^3^ School of Chemistry Beihang University Beijing 100191 China

**Keywords:** Li_2_S deposition/decomposition, lean electrolyte, lithium dendrite, lithium–sulfur battery, redox kinetics

## Abstract

Practical application of lithium–sulfur (Li–S) batteries is severely impeded by the random shuttling of soluble lithium polysulfides (LiPSs), sluggish sulfur redox kinetics, and uncontrollable growth of lithium dendrites, particularly under high sulfur loading and lean electrolyte conditions. Here, nitrogen‐doped bronze‐phase TiO_2_(B) nanosheets with oxygen vacancies (OVs) grown in situ on MXenes layers (N‐TiO_2−_ *_x_* (B)‐MXenes) as multifunctional interlayers are designed. The N‐TiO_2−_ *_x_* (B)‐MXenes show reduced bandgap of 1.10 eV and high LiPSs adsorption‐conversion‐nucleation‐decomposition efficiency, leading to remarkably enhanced sulfur redox kinetics. Moreover, they also have lithiophilic nature that can effectively suppress dendrites growth. The cell based on the N‐TiO_2−_ *_x_* (B)‐MXenes interlayer under sulfur loading of 2.5 mg cm^−2^ delivers superior cycling performance with a high specific capacity of 690.7 mAh g^−1^ over 600 cycles at 1.0 C. It still has a notable areal capacity of 6.15 mAh cm^−2^ after 50 cycles even under a high sulfur loading of 7.2 mg cm^−2^ and a low electrolyte‐to‐sulfur (E/S) ratio of 6.4 µL mg^−1^. The Li‐symmetrical battery with the N‐TiO_2−_ *_x_* (B)‐MXenes interlayer showcases a low over‐potential fluctuation with 21.0 mV throughout continuous lithium plating/stripping for 1000 h. This work offers valuable insights into the manipulation of defects and heterostructures to achieve high‐energy Li–S batteries.

## Graphical abstract

N‐doped TiO_2_(B) nanosheets are developed with abundant oxygen vacancies in situ grown on MXenes layers as multifunctional interlayer to enhance chemical trapping toward soluble LiPSs, lower Gibbs free energies for multisteps sulfur reactions, accelerate bidirectional solid Li_2_S deposition/dissolution kinetics, and suppress growth of Li dendrites.

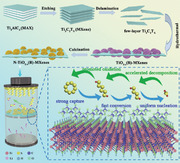

## 1 Introduction

The ever‐increasing demands for portable electronics and electric vehicles have greatly prompted the development of novel battery systems with high energy density (>400 Wh kg^−1^).^[^ ^1a–e^ ^]^ Among them, lithium–sulfur (Li–S) batteries with cost saving, remarkable theoretical energy density of 2600 Wh kg^−1^, environmental benignity, and natural abundance have been viewed as strongly competitive candidates.^[^ ^2a–e^ ^]^ However, there are still drawbacks that hinder practical application of Li–S batteries, and the common solution is to use low sulfur loading (<2.0 mg cm^−2^) and large electrolyte/sulfur ratio (E/S) (>20 µL mg^−1^).^[^ ^3^ ^]^ While this configuration may ensure reasonable battery discharge capacity, it exacerbates shuttling of soluble Li polysulfides (LiPSs) and significantly diminishes the entire energy density of battery.^[^ ^4a,b^ ^]^ High sulfur loading and lean electrolyte have been considered to be prerequisites for realizing high energy density Li–S batteries.^[^ ^5^ ^]^ However, by increasing sulfur loading and reducing low E/S ratio, the produced high‐concentration LiPSs leads to sluggish redox kinetics and uncontrollable deposition of solid Li_2_S, and exacerbates the corrosion of Li anode and the growth of dendrites, resulting in low sulfur utilization, poor cycling performance, and short battery life.^[^ ^6a,b^ ^]^

Much effort has been dedicated to overcoming these challenges associated with high sulfur loading and lean electrolyte conditions in Li–S batteries.^[^ ^7a–d^ ^]^ One effective strategy is to introduce interlayers between cathode and separator.^[^ ^8a–c^ ^]^ These interlayers, when coated on separator, could serve as barriers, which effectively confine LiPSs and protect Li anode.^[^ ^9^ ^]^ Transition‐metal oxides (TMOs) can serve as such interlayer materials due to their strong chemisorption for LiPSs/Li^+^ and catalytic effects for sulfur redox reaction.^[^ ^10a–d^ ^]^ Notably, the bronze‐phase TiO_2_ (TiO_2_(B)) shares the characteristics of TMOs and additionally promotes Li‐ion transport through open tunnels along the [010] direction.^[^ ^11a–c^ ^]^ Despite these advantages, its poor electronic conductivity and unsatisfactory catalytic activity limit its effectiveness in promoting conversion of high‐concentration LiPSs.^[^ ^12a–c^ ^]^

To enhance electrochemical activity of TiO_2_(B) interlayer, it has proven effective to integrate it with conductive materials to form heterostructure.^[^ ^13^ ^]^ Moreover, the “self‐protection” characteristic of heterostructure materials can further enhance structural stability during the long‐term cycling process.^[^ ^14a,b^ ^]^ In contrast to the carbonaceous materials, MXenes possess metallic nature and abundant functional groups, which can effectively capture LiPSs and allow in situ growth of other materials on their surface.^[^ ^15^ ^]^ For instance, Lv et al.^[^ ^9^ ^]^ designed TiO_2_‐MXene heterostructures as interlayers, which can effectively capture LiPSs and offer high catalytic activity for fast LiPSs conversion under high sulfur loading, yet nonlean electrolyte. Introducing defects into heterostructures is known to enhance electronic conductivity and offer more catalytic active sites for sulfur redox reaction.^[^ ^16a,b^ ^]^ Therefore, constructing TiO_2_(B)‐MXene heterostructures with abundant defects may simultaneously strengthen adsorption and catalytic activity toward LiPSs/Li^+^ under high sulfur loading and lean electrolyte. However, such heterostructures are rarely reported and the corresponding catalytic mechanism remains unclear.

Here, we design N‐doped TiO_2_(B) nanosheets with abundant oxygen vacancies (OVs) in situ grown on MXenes layers (N‐TiO_2−_ *_x_* (B)‐MXenes), and demonstrate that the heterostructures can lower bandgap to 1.10 eV, improve Li‐ion diffusion kinetics, enhance adsorptive, and catalytic abilities toward LiPSs, leading to promotion of sulfur redox kinetics and control of Li deposition behaviors. As a result, the cell with N‐TiO_2−_ *_x_* (B)‐MXenes interlayer under a sulfur loading of 2.5 mg cm^−2^ and an E/S ratio of 15.0 µL mg^−1^ exhibits superior long‐term cycling performance with a low‐capacity decay of 0.053% per cycle over 600 cycles at 1.0 C. The high areal capacity maintains even under high sulfur loading and lean electrolyte conditions. Additionally, the lithiophilic nature of the N‐TiO_2−_ *_x_* (B)‐MXenes facilitates extension of lithium stripping/plating cycles for over 1000 h.

## 2 Results and Discussion

### 2.1 Synthesis and Characterization of N‐TiO_2−_ *_x_* (B)‐MXenes

**Figure 1:**
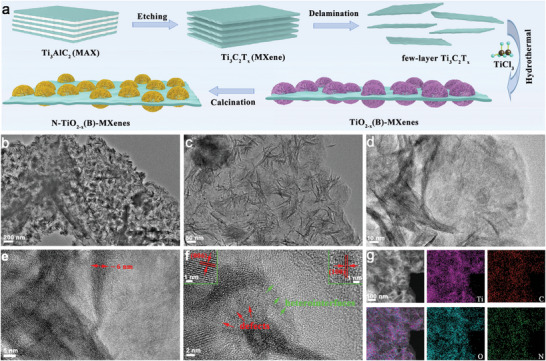
a) Schematic illustration of the N‐TiO_2−_ *_x_* (B)‐MXenes preparation process. b–e) TEM, f) HRTEM, g) HAADF‐STEM, and element mapping images of N‐TiO_2−_ *_x_* (B)‐MXenes.

The preparation process of N‐TiO_2−_ *_x_* (B)‐MXenes heterostructures is schematically depicted in **Figure** **1**a. The few‐layer MXene nanosheets were first synthesized via modified LiF/HCl mixture to extract Ti_3_AlC_2_, followed by ultrasonic stripping. As shown in Figure S1a,b (Supporting Information), the dark green MXenes suspension presents an obvious Tyndall effect and their layer thickness is ≈3 nm measured by atomic force microscopy (AFM), indicative of successful etching Ti_3_C_2_T*_x_* nanosheets with ≈1–3 layers. Subsequently, the TiO_2_(B)‐MXenes precursors were fabricated through a simple solvothermal reaction. The scanning electron microscopy (SEM) images reveal that the TiO_2_(B) nanoflowers are composed of nanosheets that grow evenly on both sides of the MXenes layers (Figure S2, Supporting Information). Finally, the TiO_2_(B)‐MXenes precursors were calcined at 350 °C under Ar atmosphere to form N‐TiO_2−_ *_x_* (B)‐MXenes heterostructures. Morphology of the resulting N‐TiO_2−_ *_x_* (B)‐MXenes maintains after calcination, and the diameter of TiO_2−_ *_x_* (B) nanoflowers is ≈150 nm, as shown in Figure S3 (Supporting Information).

Transmission electrons microscopy (TEM) images reveals that the TiO_2−_ *_x_* (B) nanosheet‐like flower petals are uniformly anchored on the MXenes layers (Figure 1b–d). The TiO_2−_ *_x_* (B) nanosheet has a thickness of ≈6 nm (Figure 1e). High‐resolution TEM (HRTEM) imaging (Figure 1f) shows that the fringes spacing of 0.27 and 0.23 nm corresponds to (100) facet of TiO_2_(B) and (006) facet of MXenes, respectively.^[^ ^9^ ^]^ Of note, there are obvious boundary area and disordered regions in crystalline lattice, suggesting successful formation of heterointerfaces and OVs. Further high‐angle annular dark‐field scanning TEM (HAADF STEM) imaging (Figure 1g) and the elemental mapping confirm the homogeneous distribution of Ti, O, C, and N elements in the N‐TiO_2−_ *_x_* (B)‐MXenes. For comparison, we also synthesized TiO_2_(B), TiO_2−_ *_x_* (B), and TiO_2−_ *_x_* (B)‐MXenes, and found that the as‐prepared TiO_2_(B) and TiO_2−_ *_x_* (B) samples show similar nanosheet spherical morphology, while their diameter (≈250 nm) is larger than that of TiO_2−_ *_x_* (B) in the N‐TiO_2−_ *_x_* (B)‐MXenes (Figures S4 and S5, Supporting Information). Such difference indicates that the MXenes layers as substrate could induce in situ growth of TiO_2_(B) on their surface and restrain the agglomeration of TiO_2_(B) nanosheets.

**Figure 2:**
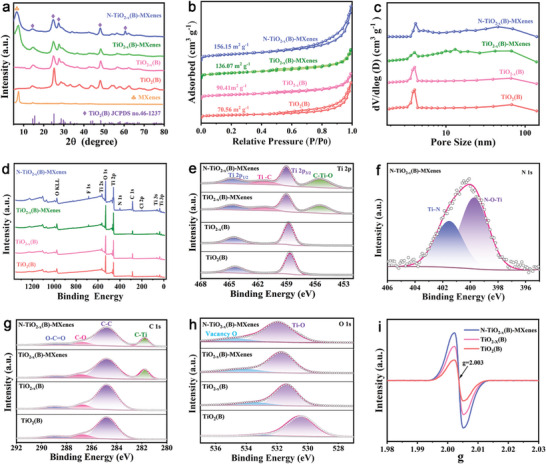
a) XRD patterns. b) N_2_ adsorption/desorption isotherms. c) Pore‐size distributions. d) Full XPS spectra. e) Ti 2p spectra. f) N 1s spectra. g) C 1s spectra. h) O 1s spectra, and i) EPR profiles of N‐TiO_2−_ *_x_* (B)‐MXenes and the reference samples.

The crystal phases of different samples were measured by X‐ray diffraction (XRD). As displayed in XRD patterns of Ti_3_AlC_2_ and MXenes (Figure S6, Supporting Information), the appearance of the Ti_3_C_2_T*_x_* characteristic peak ((002)) at ≈7° and the disappear of Ti_3_AlC_2_ diffraction peaks demonstrate the successful etching of Al layer from MXA phase.^[^ ^17^ ^]^ As for the TiO_2_(B), TiO_2−_ *_x_* (B), TiO_2−_ *_x_* (B)‐MXenes, and N‐TiO_2−_ *_x_* (B)‐MXenes, their main diffraction peaks center at 14.2°, 24.9°, and 48.5°, corresponding to the (001), (110), and (020) crystal planes labeled (◇).^[^ ^18^ ^]^ These peaks could be assigned to the bronze‐phase TiO_2_ (JCPDS No. 46‐1237) and other peaks labeled (♧) belongs to the MXenes (**Figure** **2**a).^[^ ^19^ ^]^ Besides, N_2_ adsorption–desorption measurement was used to perform the specific surface area and pore size of these samples. As observed in Figure 2b,c, the N‐TiO_2−_ *_x_* (B)‐MXenes possess hierarchical porous structure with the largest specific surface area of 156.15 cm^2^ g^−1^, which is beneficial to provide more adsorption and catalytic active sites toward soluble LiPSs.

The X‐ray photoelectron spectroscopy (XPS) was used to investigate the chemical composition and valence states for all samples. As depicted in Figure 2d, the N 1s signal was seen only in the full XPS spectra of N‐TiO_2−_ *_x_* (B)‐MXenes, manifesting the successful doping of N‐atom. In the high‐resolution Ti 2p spectra (Figure 2e) of N‐TiO_2−_ *_x_* (B)‐MXenes and TiO_2−_ *_x_* (B)‐MXenes, the peaks could be divided into four pairs of peaks at 455.7, 458.8, 461.8, and 464.6 eV, corresponding to C‐Ti‐O, Ti 2p_3/2_, Ti‐C, and Ti 2p_1/2_, respectively.^[^ ^9^ ^]^ From the N 1s spectra of N‐TiO_2−_ *_x_* (B)‐MXenes (Figure 2f), the peaks located at 399.8 and 401.8 eV are attributed to N‐O‐Ti and Ti‐N, respectively, further supporting that the N‐atoms are doped into the heterostructures and occupy the position of O.^[^ ^20^ ^]^ The characteristic bonds (Ti‐C, C‐C, and et al.) in MXenes were well seen in high‐resolution C 1s spectra (Figure 2g).^[^ ^21^ ^]^ As shown in the high‐resolution O 1s spectra (Figure 2h), the obviously fitted peaks of vacancy O in N‐TiO_2−_ *_x_* (B)‐MXenes, TiO_2−_ *_x_* (B)‐MXenes, and TiO_2−_ *_x_* (B) reveal the existence of OVs on their surface. The O‐Ti peaks (≈531.5 eV) for the N‐TiO_2−_ *_x_* (B)‐MXenes shifted to lower energy states in comparison to that for TiO_2_(B), probably causing by charge redistribution closely related to the existence of OVs, N‐doping and heterointerfaces.^[^ ^22^ ^]^ Moreover, electron paramagnetic resonance (EPR) measurements were carried out to further investigate the defect properties of these samples, as presented in Figure 2i. The strong signals at *g* = 2.003 for the N‐TiO_2−_ *_x_* (B)‐MXenes, TiO_2−_ *_x_* (B)‐MXenes, and TiO_2−_ *_x_* (B) could be attributed to the unpaired electrons trapped via OVs.^[^ ^23a,b^ ^]^ These results verify the successful introduction of OVs and N‐atom, and the formation of heterointerfaces in the N‐TiO_2−_ *_x_* (B)‐MXenes.

### 2.2 Band Structures and Adsorption Ability

**Figure 3:**
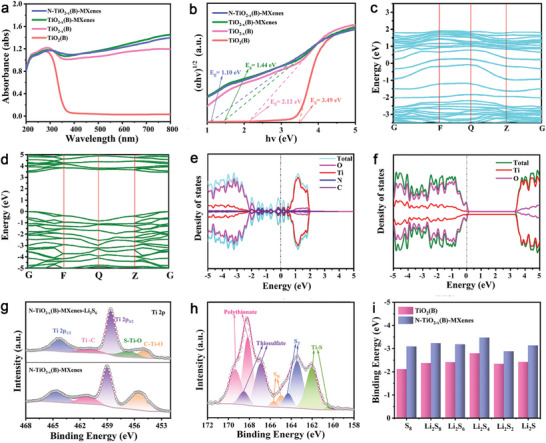
a) UV−vis absorption spectra and b) corresponding Kubelka–Munk plots of all samples. Calculated band structures and DOS of c,e) N‐TiO_2−_ *_x_* (B)‐MXenes and d,f) TiO_2_(B). g) Ti 2p XPS spectra for the N‐TiO_2−_ *_x_* (B)‐MXenes before and after Li_2_S_6_ adsorption. h) S 2p XPS spectra for the N‐TiO_2−_ *_x_* (B)‐MXenes after Li_2_S_6_ adsorption. i) Calculated binding energies of sulfur species on the surface of N‐TiO_2−_ *_x_* (B)‐MXenes and TiO_2_(B).

In UV−vis absorption spectra (**Figure** **3**a), the absorption sharp edge for the N‐TiO_2−_ *_x_* (B)‐MXenes exhibits the most pronounced redshift compared to the other three samples. Utilizing the Kubelka–Munk function, the derived bandgap values (Figure 3b) reveal that the N‐TiO_2−_ *_x_* (B)‐MXenes have a considerably reduced bandgap of 1.10 eV, in stark contrast to the 3.49 eV bandgap of TiO_2_(B). This significant bandgap narrowing in N‐TiO_2−_ *_x_* (B)‐MXenes is favorable to enhance electronic conductivity.^[^ ^24^ ^]^ Density functional theory (DFT) simulations were performed to deeply investigate the difference of band structures in N‐TiO_2−_ *_x_* (B)‐MXenes and TiO_2_(B). As seen in the calculated band structures derived from the optimized structural models (Figure S7, Supporting Information), the integration into heterostructures along with the incorporation of OVs and N‐atoms significantly alters the electronic properties of TiO_2_(B). This modification results in a nearly negligible bandgap energy for N‐TiO_2−_ *_x_* (B)‐MXenes, while, bandgap energy for TiO_2_(B) is much larger (Figure 3c,d). Furthermore, the calculated density of states (DOS) for the heterostructures (Figure 3e) is closer to the Fermi level in contrast to TiO_2_(B) (Figure 3f), further implying favorable charge transfer on the surface of N‐TiO_2−_ *_x_* (B)‐MXenes heterostructures. These findings elucidate that the integration of TiO_2_(B) with MXenes, alongside the incorporation of OVs and N‐atoms, adeptly narrows the bandgap while simultaneously enhancing electronic conductivity.

To assess the adsorption capacities of the four samples for soluble LiPSs, visual adsorption experiments were arranged through adding identical amount of samples into the Li_2_S_6_ solution. After standing for 5 h, the color of Li_2_S_6_ solution treated with N‐TiO_2−_ *_x_* (B)‐MXenes and TiO_2−_ *_x_* (B)‐MXenes became almost transparent while that with TiO_2−_ *_x_* (B) and TiO_2_(B) was pale yellow and brown (Figure S8a, Supporting Information), respectively. The corresponding UV–vis absorption spectra (Figure S8b, Supporting Information) of these Li_2_S_6_ solution treated with samples also verify the strongest adsorption ability of N‐TiO_2−_ *_x_* (B)‐MXenes toward soluble Li_2_S_6_. To delve deeper into the interactions between N‐TiO_2−_ *_x_* (B)‐MXenes and LiPSs, XPS analyses were conducted after soaking for 12 h under Li_2_S_6_‐solution. As depicted in Figure 3g, the Ti 2p peaks of N‐TiO_2−_ *_x_* (B)‐MXenes exhibit marked shifts post Li_2_S_6_ adsorption. A newly emerged peak at 456.8 eV is postulated to represent the S*─*Ti*─*O bond, resulting from the interaction between TiO_2_ and LiPSs. The detection of bridging sulfur (S_B_^0^), terminal sulfur (S_T_^−1^), and Ti*─*S bonds in the S 2p spectra (Figure 3h) also unequivocally substantiates the trapping of LiPSs on the surface of N‐TiO_2−_ *_x_* (B)‐MXenes.^[^ ^25^ ^]^

Due to the inability of commercial Celgard polypropylene (PP) separators, characterized by their large pores (Figure S9a, Supporting Information), to effectively block the LiPSs shuttling, a coating of N‐TiO_2−_ *_x_* (B)‐MXenes was applied to their surface. As depicted in Figure S9b (Supporting Information), the N‐TiO_2−_ *_x_* (B)‐MXenes modified PP separator still preserves its flexibility even after being folded. From the cross‐sectional SEM image, the thickness of the coating layer is ≈5 µm (Figure S10a, Supporting Information). For comparative purposes, separators were coated with the other three samples, each employing an equivalent mass for the coating. The permeability experiments of LiPSs were tested by using H‐shaped glass cells with different interlayers. As observed in Figure S10b (Supporting Information), almost no yellow LiPSs cross the N‐TiO_2−_ *_x_* (B)‐MXenes interlayer even after 24 h, while the solution using other interlayers presents varying degrees of yellow color, implying that the N‐TiO_2−_ *_x_* (B)‐MXenes effectively immobilize LiPSs. DFT calculations were performed to in‐depth simulate the binding of sulfur species on different surfaces. The optimized configurations and corresponding calculated results were shown in Figure 3i; and Figure S11 (Supporting Information), severally. It could be seen that the binding energies of sulfur species on the surface of N‐TiO_2−_ *_x_* (B)‐MXenes (S_8_ −3.09 eV, Li_2_S_8_ −3.23 eV, Li_2_S_6_ −3.18 eV, Li_2_S_4_ −3.47 eV, Li_2_S_2_ −2.88 eV, and Li_2_S −3.13 eV) are all larger compared to those on TiO_2_(B) surface (S_8_ −2.11 eV, Li_2_S_8_ −2.37 eV, Li_2_S_6_ −2.41 eV, Li_2_S_4_ −2.79 eV, Li_2_S_2_ −2.34 eV, and Li_2_S −2.72 eV). Hence, the conjunction of experimental tests and DFT calculations conclusively demonstrate that N‐TiO_2−_ *_x_* (B)‐MXenes possess superior adsorption capacity for both soluble LiPSs and solid Li_2_S_2_/Li_2_S.

### 2.3 Catalytic Effects toward LiPSs and Li_2_S

**Figure 4:**
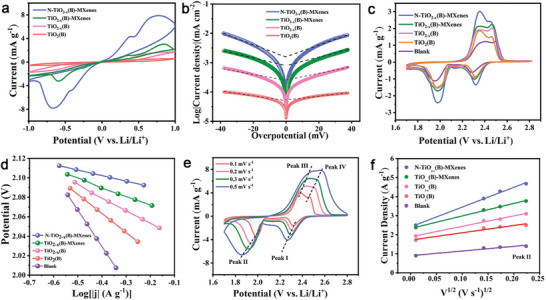
a) CV profiles of symmetric cells at a scanning rate of 0.5 mV s^−1^. b) Tafel plots of these symmetric cells. c) CV profiles of asymmetric at a scanning rate of 0.1 mV s^−1^ and d) Tafel plots derived from Peak II of the CV profiles. e) CV curves of the cells with N‐TiO_2−_ *_x_* (B)‐MXenes interlayers at various scanning rates. f) Peak II in CV curves versus the square root of scan rates.

Symmetric/asymmetric cell tests and Li_2_S precipitation/dissociation assessments were systematically executed to ascertain the catalytic prowess of N‐TiO_2−_ *_x_* (B)‐MXenes in facilitating the conversion of LiPSs and Li_2_S. Symmetric cells containing Li_2_S_6_ electrolyte were assembled for conducting cyclic voltammetry (CV) tests at a scanning rate of 0.5 mV s^−1^ in a voltage window of −1.0 to 1.0 V. As illustrated in **Figure** **4**a, the CV profile of the TiO_2−_ *_x_* (B)‐MXenes symmetric cell presents two sets of redox peaks, arising from two steps in the conversion process of LiPSs (Li_2_S_6_ ↔ Li_2_S_4_ ↔ Li_2_S).^[^ ^26^ ^]^ Within the examined CV profiles, the N‐TiO_2−_ *_x_* (B)‐MXenes symmetric cell exhibits a higher response current, reduced polarization voltage, and sharper redox peaks relative to those of the other cells. This suggests that the incorporation of OVs, the formation of heterostructures, and the doping of N‐atoms collectively contribute to the accelerated redox kinetics for LiPSs. When subjected to large scanning rate of 50 mV s^−1^, the CV profile (Figure S12, Supporting Information) of the N‐TiO_2−_ *_x_* (B)‐MXenes symmetric cell retains clear redox peaks with minimal potential gap, unlike the less distinct or dulled peaks observed in CV profiles of the other cells. This underscores the superior catalytic functionality of N‐TiO_2−_ *_x_* (B)‐MXenes in promoting the conversion of LiPSs under high current conditions.^[^ ^27^ ^]^ Additionally, the lowest charge transfer resistance for N‐TiO_2−_ *_x_* (B)‐MXenes electrode in the electrochemical impedance spectroscopy (EIS) curves (Figure S13, Supporting Information) also further corroborates the enhanced catalytic efficacy Tafel plots derived from these symmetric cell tests (Figure 4b) reveal that the N‐TiO_2−_ *_x_* (B)‐MXenes cell generates a higher response current for both reduction and oxidation process. Crucially, the calculated Tafel slope is lower, and the exchange current density is notably higher (4.28 × 10^−2^ mA cm^−2^) for the N‐TiO_2−_ *_x_* (B)‐MXenes cell in comparison to the TiO_2−_ *_x_* (B)‐MXenes (3.25 × 10^−2^ mA cm^−2^), TiO_2−_ *_x_* (B) (2.56 × 10^−2^ mA cm^−2^), and TiO_2_(B) (2.28 × 10^−2^ mA cm^−2^), which is indicative of a beneficial impact on accelerating the redox kinetics of LiPSs.

The CV measurements of asymmetric cells with different interlayers for the first cycle at a scanning rate of 0.1 mV s^−1^ were conducted in the range of 1.7−2.8 V, as shown in Figure 4c. In these profiles, two cathodic peaks observed at ≈2.31 V (Peak I) and 2.04 V (Peak II) are attributed to the transformation of S_8_ into LiPSs and subsequently into Li_2_S.^[^ ^28^ ^]^ Additionally, two anodic peaks at around 2.33 V (Peak III) and 2.43 V (Peak IV) are indicative of the reverse conversion of Li_2_S back to S_8_.^[^ ^29^ ^]^ In comparison to the cell with TiO_2−_ *_x_* (B)‐MXenes, TiO_2−_ *_x_* (B), or TiO_2_(B) interlayers, the cell with N‐TiO_2−_ *_x_* (B)‐MXenes interlayer exhibits a significantly smaller polarization and a higher peak current. This suggests that the N‐TiO_2−_ *_x_* (B)‐MXenes effectively enhance electron transfer and catalytic activity during the sulfur redox reactions. The corresponding Tafel plots derived from Peak II of the CV curves were also calculated to quantify the catalytic activities. As illustrated in Figure 4d, the Tafel slopes for the cell with N‐TiO_2−_ *_x_* (B)‐MXenes interlayer were determined to be 24.6 mV dec^−1^, notably lower than those with TiO_2−_ *_x_* (B)‐MXenes (32.8 mV dec^−1^), TiO_2−_ *_x_* (B) (46.6 mV dec^−1^), and TiO_2_(B) (55.4 mV dec^−1^) interlayers, indicating the enhancement of catalytic activity for N‐TiO_2−_ *_x_* (B)‐MXenes in the LiPSs reduction process.

CV measurements were further conducted on these cells across a range of scanning rates from 0.1 to 0.5 mV s^−1^ to investigate the diffusion properties of Li‐ions, with results as shown in Figure 4e; and Figure S14 (Supporting Information). The current density of both cathodic and anodic peaks for the cells in the CV curves exhibits a linear relationship with the square root of the scanning rates. This relationship aligns with the classical Randles–Sevcik equation (*I*_p_ = (2.69 × 10^5^).*n* ^1.5^ *SD* ^0.5^ *C*ν^0.5^). In this equation, *I*_p_ represents the peak current density, *n* denotes the number of electrons involved in the charge transfer, *S* is the electrode area, *D* stands for the Li‐ion diffusion coefficient, *C* signifies the concentration of Li ions, and ν is the potential scanning rate. The slopes derived from the CV curves are directly proportional to Li‐ion diffusion rates.^[^ ^30^ ^]^ The cell with N‐TiO_2−_ *_x_* (B)‐MXenes interlayer exhibits the steepest slopes (Figure 4f; and Figure S15 (Supporting Information)), indicating superior Li‐ion diffusivity on the surface of N‐TiO_2−_ *_x_* (B)‐MXenes, surpassing that of other samples.

**Figure 5:**
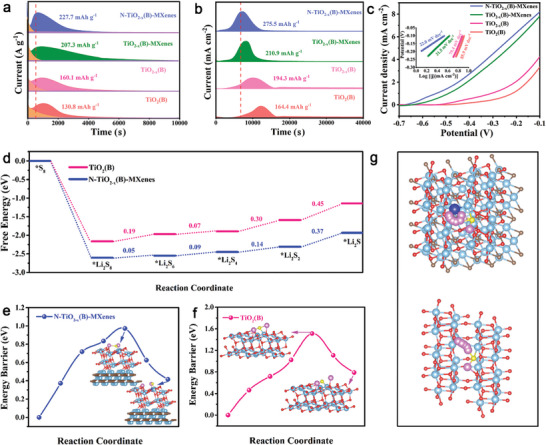
a) Potentiostatic discharge and b) potentiostatic charge profiles, c) LSV profiles and corresponding Tafel plots of different samples (inset in Figure 5c). d) Calculated Gibbs free energy for the different sulfur species. e,f) decomposition barriers of Li_2_S (insets: the transition and final structures) and g) corresponding dissociation path on N‐TiO_2−_ *_x_* (B)‐MXenes and TiO_2_(B).

The nucleation and growth of Li_2_S are crucial aspects of the liquid‐solid conversion process of LiPSs, contributing to three‐quarters of the theoretical battery capacity. As shown in the potentiostatic discharge curve (**Figure** **5**a), the response time for Li_2_S nucleation and the peak current in the N‐TiO_2−_ *_x_* (B)‐MXenes (694 s and 227.3 mAh g^−1^) are significantly earlier and higher, respectively, compared to those in TiO_2−_ *_x_* (B)‐MXenes (965 s and 207.3 mAh g^−1^), TiO_2−_ *_x_* (B) (1147 s and 160.1 mAh g^−1^), and TiO_2_(B) (1372 s and 130.8 mAh g^−1^). The Li_2_S nucleation capacity calculated for N‐TiO_2−x_(B)‐MXenes, at 275.5 mAh g^−1^, is notably higher than those of TiO_2−_ *_x_* (B)‐MXenes (210.9 mAh g^−1^), TiO_2−_ *_x_* (B) (194.3 mAh g^−1^), and TiO_2_(B) (160.4.4 mAh g^−1^). Meanwhile, it also shows a much higher current peak and larger dissolution capability of Li_2_S (275.5 mAh g^−1^) compared to the other three electrodes during the charge process (Figure 5b). Furthermore, linear sweep voltammetry (LSV) analysis was conducted to assess the oxidation of Li_2_S, as exhibited in Figure 5c. The TiO_2−_ *_x_* (B)‐MXenes electrode displays the lowest onset potential and the highest current density response among these electrodes, suggesting a reduced energy barrier for Li_2_S oxidation. This conclusion is corroborated by Tafel plot data (inset in Figure 5c), where the N‐TiO_2−_ *_x_* (B)‐MXenes electrode exhibits the smallest Tafel slope of 22.0 mV dec^−1^, in contrast to TiO_2−_ *_x_* (B)‐MXenes (31.5 mV dec^−1^), TiO_2−_ *_x_* (B) (79.4 mV dec^−1^), and TiO_2_(B) (85.9 mV dec^−1^). These results highlight the superior catalytic activity of N‐TiO_2−_ *_x_* (B)‐MXenes in promoting deposition/decomposition of Li_2_S process.

DFT calculations were further employed to deeply investigate the energy dynamics of the multistep reaction process in LSBs at the molecular level. The calculated Gibbs free energy (ΔG) values for the reduction process of S_8_ to different sulfur species (Li_2_S_8_, Li_2_S_6_, Li_2_S_4_, Li_2_S_2_, Li_2_S) on different surfaces are presented in Figure 5d. The conversion from Li_2_S_2_ to Li_2_S (solid → solid) is recognized as the rate‐determining step (RDS) due to the slowest kinetics.^[^ ^31^ ^]^ In particular, N‐TiO_2−_ *_x_* (B)‐MXenes exhibits the lowest ΔG value for each step reaction, even for the RDS (0.37 eV), in comparison to TiO_2_(B) (0.45 eV), indicating more thermodynamically favorable conversions of LiPSs and the nucleation of Li_2_S on N‐TiO_2−_ *_x_* (B)‐MXenes. Moreover, the dissociation behaviors of Li_2_S into LiS and Li^+^ on different surfaces were also investigated during the charging process. As indicated in Figure 5e–g, the decomposition energy of Li_2_S on N‐TiO_2−_ *_x_* (B)‐MXenes (0.98 eV) is lower than that on TiO_2_(B) (1.52 eV). This suggests enhanced decomposition kinetics of Li_2_S on the N‐TiO_2−_ *_x_* (B)‐MXenes surface. Collectively, these experimental and theoretical findings demonstrate that the synergistic effect between N‐TiO_2−_ *_x_* (B) and MXenes significantly enhances the chemisorption and promote the conversion reactions for LiPSs, as well as expedite the nucleation/decomposition process of Li_2_S.

### 2.4 Battery Performance

**Figure 6:**
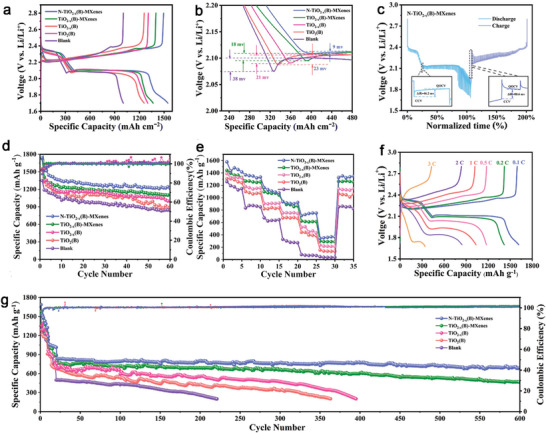
a) Charge–discharge profiles. b) Enlarged part of the charge‐discharge profiles. c) GITT profiles. d) cycling performances at 0.1 C, and e) rate capabilities of the cells with different modified separators. f) Charge–discharge profiles of the cell with N‐TiO_2−_ *_x_* (B)‐MXenes@PP at various current rates. g) Long‐term cycling performances of these cells at 1.0 C.

Battery performances were measured using coin cells constructed with a sulfur cathode and various interlayers, each under a sulfur loading of ≈2.5 mg cm^−2^ and an E/S ratio of 15.0 µL mg^−1^. The sulfur content in the as‐prepared sulfur cathode was ≈68.3 wt%, as determined by thermogravimetric analysis (TGA), as depicted in Figure S16 (Supporting Information). As displayed in **Figure** **6**a, the galvanostatic charge/discharge profiles for all cells show typical plateaus of LSBs, aligning with observations from the CV analysis. The cell with N‐TiO_2−_ *_x_* (B)‐MXenes interlayer delivers higher initial specific discharge/charge capacities of 1546/1535 mAh g^−1^ and a lower overpotential of polarization potential of 178 mV. In comparison, the performance metrics of other cells, specifically those with TiO_2−_ *_x_* (B)‐MXenes (1479/1453 mAh g^−1^, 189 mV), TiO_2−_ *_x_* (B) (1355/1321 mAh g^−1^, 201 mV), or TiO_2_(B) interlayer (1313/1289 mAh g^−1^, 213 mV), reveal lower capacities and higher polarization potentials. Notably, the prominent valley between the high and low discharge plateau represents the “nucleation points of Li_2_S.” The potential difference between the tangential of the low discharge plateau and nucleation point offers a quantifiable means to explore the reaction kinetics associated with the nucleation of Li_2_S.^[^ ^32^ ^]^ Among the evaluated cells, the cell with N‐TiO_2−_ *_x_* (B)‐MXenes interlayer distinguishes itself by presenting the lowest nucleation overpotential at 9 mV, indicative of the most rapid Li_2_S precipitation at the interface, as shown in Figure 6b. Moreover, the ratio of the capacity at the low‐voltage plateau (*Q*_L_) to that at the high‐voltage plateau (*Q*_H_) serves as a marker for the conversion efficiency from liquid to solid phases (LiPSs to Li_2_S_2_/Li_2_S). The cell with N‐TiO_2−_ *_x_* (B)‐MXenes@PP exhibits a higher *Q*_L_/*Q*_H_ ratio compared to those cells (Figure S17, Supporting Information), highlighting superior conversion kinetics of LiPSs. This is further corroborated by the lowest internal resistances recorded at the nucleation and activation points for Li_2_S in the cell, validated via the galvanostatic intermittent titration technique (GITT) results shown in Figure 6c; and Figure S18 (Supporting Information). Moreover, the calculated D_Li_^+^ value of the cell with N‐TiO_2−_ *_x_* (B)‐MXenes interlayer according to the GITT curves is higher than others during the discharge and charge processes (Figure S19, Supporting Information). The cycling performances of these cells at 0.1 C (1.0 C = 1675 mA g^−1^) are illustrated in Figure 6d. When compared to the cells with TiO_2−_ *_x_* (B)‐MXenes (1115.8 mAh g^−1^), TiO_2−_ *_x_* (B) (998.8 mAh g^−1^), and TiO_2_(B) interlayers (878.6 mAh g^−1^), that with N‐TiO_2−_ *_x_* (B)‐MXenes interlayer achieves a higher specific capacity of 1216.7 mAh g^−1^ and a retention rate of 90% over 60 cycles. Meanwhile, the cell with N‐TiO_2−_ *_x_* (B)‐MXenes interlayer reaches a good rate capability, with high specific capacities of 1430.9, 1355.6, 1129.4, 934.8, 709.0, and 380.9 mAh g^−1^ at 0.1, 0.2, 0.5, 1.0, 2.0, and 3.0 C, respectively (Figure 6e). This rate performance is superior to that of TiO_2−_ *_x_* (B)‐MXenes (1324.5, 1267.9, 1078.3, 890.6, 622.9, and 320.5 mAh g^‐1^), TiO_2−_ *_x_* (B) (1221.4, 1140.3, 900.4, 752.6, 530.5, and 265.2 mAh g^−1^) and TiO_2_(B) interlayers (1184.4, 1076.1, 820.4, 672.6, 441.1, and 225.2 mAh g^−1^) at the corresponding current rates. The capacity of the cell with N‐TiO_2−_ *_x_* (B)‐MXenes interlayer still maintains 1327.3 mAh g^−1^ while returning to 0.1 C, proving outstanding reversibility and stability. On the other side, its charge/discharge voltage curves also display distinct plateaus even at 3.0 C, unlike the pronounced polarization observed in other cells (Figure 6f; and Figure S20, Supporting Information). The EIS analysis of these cells before and after cycling were examined. As shown in Figure S21 (Supporting Information), the N‐TiO_2−_ *_x_* (B)‐MXenes cell delivers smaller charge transfer impedance and Warburg resistance than the others, indicating the accelerated LiPSs conversion kinetics. The long‐term cycling performance of these cells at 1.0 C was meticulously assessed, as depicted in Figure 6g. Following an initial activation at 0.1 C, the cell with N‐TiO_2−_ *_x_* (B)‐MXenes interlayer shows an initial specific capacity of 1063.0 mAh g⁻¹. This performance surpasses those observed in the cells with TiO_2−_ *_x_* (B)‐MXenes (1008.1 mAh g^−1^), TiO_2−_ *_x_* (B) (963.8 mAh g^−1^), and TiO_2_(B) interlayers (910.5 mAh g^−1^). Remarkably, even after 600 cycles, this cell sustains a high reversible specific capacity of 690.7 mAh g^−1^, accompanied by a low‐capacity decay rate of 0.053% per cycle. In stark contrast, the other cells exhibit poor cycling stability.

**Figure 7:**
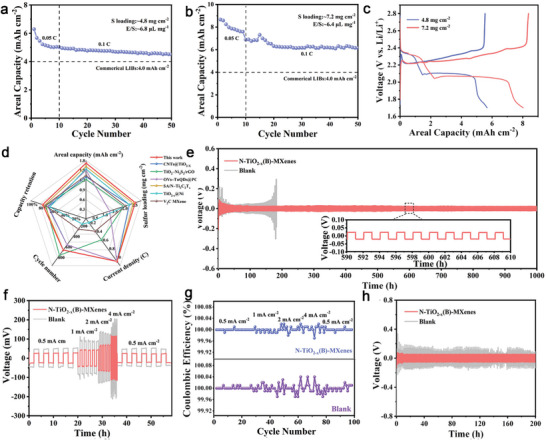
a) Cycling performances of the cell with N‐TiO_2−_ *_x_* (B)‐MXenes interlayer under a high sulfur loading of 4.8 mg cm^−2^ and b) under a high sulfur loading of 7.2 mg cm^−2^. c) Charge–discharge profiles under high sulfur loadings. d) Battery performances based on similar samples. e) Galvanostatic cycling of the symmetric cells at 0.5 mA cm^−2^ and 1.0 mAh cm^−2^ and f) rate performances at various current densities. g) Corresponding Coulombic efficiency of Li||Li symmetric cells with different electrodes at different current densities. h) Galvanostatic cycling of the symmetric cells at 2.0 mA cm^−2^ and 1.0 mAh cm^−2^.

High sulfur loading and lean electrolyte conditions are crucial benchmarks for the commercial viability of Li−S battery.^[^ ^33a,b^ ^]^ Remarkably, under a sulfur loading of 4.8 mg cm⁻^2^ and low E/S ratio of 6.8 µL mg⁻¹, the cell with N‐TiO_2−_ *_x_* (B)‐MXenes@PP maintains a high areal capacity of 4.5 mAh cm^−2^ after 50 cycles at 0.1 C (**Figure** **7**a). Upon increasing the sulfur loading to 7.2 mg cm^−2^ and decreasing the E/S ratio to 6.4 µL mg^−1^, the galvanostatic charge/discharge profiles of this cell still exhibit the characteristic discharging/charging plateau, achieving an elevated areal capacity of 6.15 mAh cm⁻^2^ after 50 cycles at 0.1 C, detailed in Figure 7b,c. Such excellent cycling performance and rate capability underscored by the cell with N‐TiO_2−_ *_x_* (B)‐MXenes@PP highlights the improved sulfur redox kinetics facilitated by a synergistic interplay among N‐doping, OVs, and dense heterointerfaces. These advancements testify the N‐TiO_2−_ *_x_* (B)‐MXenes' status as modified separators, outperforming many recently reported works, as summarized in Figure 7d; and Table S1 (Supporting Information).^[^ ^1^, ^34^ ^]^

### 2.5 Inhibition of Lithium Dendrites

Noteworthy that the electrolyte wettability of the interlayers is a critical factor for the Li‐ion interfacial transport and reversible Li stripping/plating. The N‐TiO_2−_ *_x_* (B)‐MXenes interlayer exhibits smaller contact angle of ≈12.07° compared with TiO_2−_ *_x_* (B)‐MXenes (≈14.14°), TiO_2−_ *_x_* (B) (≈18.88°), TiO_2_(B) (≈20.56°), and pristine PP (≈32.99°), indictive of a good electrolyte wettability (Figure S22, Supporting Information). To explore the influence of various materials on Li dendrite formation, cells were disassembled after cycling. The SEM images reveal that the Li anode surface in the cell with N‐TiO_2−_ *_x_* (B)‐MXenes interlayer is markedly smoother and exhibits fewer dendrites compared to the anode in blank cell, as shown in Figure S23 (Supporting Information). Moreover, the symmetric cells (Li||Li) were further assembled, and their stripping/plating process was tested at 0.5 mA cm^−2^ and 2 mAh cm^−2^. When compared to the higher overpotential observed in bare Li||Li cell, the Li||Li cell with N‐TiO_2−_ *_x_* (B)‐MXenes interlayer displays a lower voltage hysteresis of ≈21 mV over 1000 h, as delineated in Figure 7e. Furthermore, this cell maintains acceptable overpotentials of 25, 41, 62, and 112 mV (Figure 7f) along with a stable coulombic efficiency (Figure 7g), at escalating current densities of 0.5, 1.0, 2.0, and 4.0 mA cm⁻^2^, respectively. Moreover, more stable cycling performance over 200 h at a high density of 2 mA cm^−2^ and 2 mAh cm^−2^ can be realized by the Li||Li cell with N‐TiO_2−_ *_x_* (B)‐MXenes interlayer (Figure 7h). As for bare Li||Li cell, a short‐circuit phenomenon could be observed after 170 h, which attributed to the heterogeneous Li deposition and unrestrained growth of dendrite. EIS analysis (Figure S24, Supporting Information) of the two cells, both before and after cycling was conducted. The Li||Li cell with N‐TiO_2−_ *_x_* (B)‐MXenes interlayer presents lower resistance compared to the bare Li||Li cell. This is indicative of enhanced wettability of the electrolyte and superior kinetics for Li stripping/plating. These results suggest that the lithiophilic surface of N‐TiO_2−_ *_x_* (B)‐MXenes plays a critical role in effectively regulating the nucleation and growth behavior of Li, thereby facilitating flat and uniform Li deposition.

## 3 Conclusion

We have successfully constructed N‐TiO_2−_ *_x_* (B)‐MXenes with abundant OVs and heterointerfaces as multifunctional interlayer in Li−S batteries, which effectively modifies both sulfur conversion kinetics and Li stripping/plating behaviors. The integrating N‐TiO_2−_ *_x_* (B) with MXenes show more active sites for LiPSs adsorption and Li_2_S nucleation/decomposition, which promote electron transport and ion diffusion, thereby enhancing overall sulfur redox kinetics. Moreover, the large lithiophilic surface of N‐TiO_2−_ *_x_* (B)‐MXenes effectively tunes the Li plating/stripping behaviors to suppress the growth of Li dendrites. As a result, the cell with the N‐TiO_2−_ *_x_* (B)‐MXenes interlayer demonstrates a remarkable long‐term cycling stability under a sulfur loading of 2.5 mg cm⁻^2^ and a high specific capacity of 690.7 mAh g^−1^ at 1.0 C over 600 cycles. Even under the challenging conditions of a high sulfur loading of 7.2 mg cm^−2^ and a low E/S ratio of 6.4 µL mg^−1^, the cell still achieves an areal capacity of 6.15 mAh cm^−2^ after 50 cycles. Additionally, Li||Li cells upon the N‐TiO_2−_ *_x_* (B)‐MXenes interlayer exhibit exceptional stability and a low overpotential fluctuation of 21 mV over 1000 h during the continuous lithium plating/stripping processes. This work offers a new insight into creating multifunctional materials to achieve high‐performance energy storage systems.

## 4 Experimental Section

### Preparation of MXenes

Few‐layered Ti_3_C_2_T*_x_* MXenes nanosheets were produced through a LiF/HCl exfoliation method. Initially, 1.6 g of LiF powder, and 1.0 g of commercial MAX (Ti_3_AlC_2_) powder were combined in 20.0 mL of 9.0 m HCl and maintained at 40.0 °C for 24.0 h. Subsequently, the resulting precipitates underwent centrifugal washing with deionized water until achieving a pH level of at least 6 to obtain the multilayer MXenes. The resultant multilayer MXenes precipitates were then dispersed in 70 mL deionized water and treated with ultrasonication (240 W of power) for 60 min, followed by centrifugation at 3500 rpm for 60 min. The supernatant was collected to obtain the colloidal solution of few‐layered Ti_3_C_2_T*_x_* MXenes.

### Preparation of TiO_2_(B) and TiO_2−_ *_x_* (B)

1.0 mL of titanium trichloride (TiCl_3_), 30.0 mL of ethylene glycol (EG), and 1.0 mL of deionized water were combined in a 50.0 mL beaker and magnetically stirred for 1.0 h. The mixture was then transferred to a 100.0 mL Teflon‐lined autoclave and heated at 150.0 °C for 24.0 h. Postreaction, TiO_2_(B) was harvested through centrifugal washing with deionized water and ethanol. The obtained precursor underwent calcination at 350.0 °C for 2.0 h under Ar atmosphere to produce TiO_2−_ *_x_* (B).

### Preparation of N‐TiO_2−_ *_x_* (B)‐MXenes

Specifically, 1.0 mL of TiCl_3_, 30.0 mL of EG, 1.0 mL of deionized water, and 3.0 mL of a Ti_3_C_2_T*_x_* solution (10 mg mL⁻¹) were combined in a 50.0 mL beaker and subjected to magnetic stirring for 1.0 h. Following this, the mixture was transferred to a 100.0 mL Teflon‐lined autoclave and heated at 150.0 °C for 24.0 h. Subsequent to the reaction, centrifugation was employed for washing the product with deionized water and ethanol to yield TiO_2_(B)‐MXenes. The obtained precursor was calcined in the presence of urea at 350.0 °C for 2.0 h in a tube furnace under Ar atmosphere, resulting in N‐TiO_2−_ *_x_* (B)‐MXenes. As part of the control experiments, TiO_2−_ *_x_* (B)‐MXenes were prepared using the same procedure but without the addition of urea.

## Conflict of Interest

The authors declare no conflict of interest.

## Supporting information

Supporting Information

## Data Availability

The data that support the findings of this study are available from the corresponding author upon reasonable request.
