# Supplementary material for: Nitrogen‐Doped TiO2− x (B)/MXene Heterostructures for Expediting Sulfur Redox Kinetics and Suppressing Lithium Dendrites
Source: Adv Sci (Weinh). 2024 Jul 23;11(36):2406475. doi: 10.1002/advs.202406475 (PMC11423229; doi:10.1002/advs.202406475)
Supplement: Supplementary file 1 — Supporting Information [file ADVS-11-2406475-s001.docx]

Supporting Information

**Nitrogen-doped TiO_2-x_(B)/MXene Heterostructures as Interlayer for Expediting Sulfur Redox Kinetics and Suppressing Lithium Dendrites**

*Mengmeng Zhen, Xiaoyu Wang, Qihang Yang, Zihang Zhang, Zhenzhong Hu*, Zhenyu Li*, and Zhongchang Wang**

**Table of contents**

[**Experimental section** 3](#_Toc166595652)

[**Supporting Figures and Tables** 7](#_Toc166595653)

[**Figure S1.** (a) The Tyndall effect of the MXenes solution, (b) AFM images of as-prepared few layered Ti_3_C_2_T_x_ MXenes nanosheets. 7](#_Toc166595654)

[**Figure S2.** SEM images of TiO_2_(B)-MXenes precursors. 7](#_Toc166595655)

[**Figure S3.** SEM images of as-prepared N-TiO_2-X_(B)-MXenes heterostructures. 7](#_Toc166595656)

[**Figure S4.** SEM images of TiO_2_(B) precursors. 8](#_Toc166595657)

[**Figure S5.** SEM images of TiO_2-X_(B) precursors. 8](#_Toc166595658)

[**Figure S6.** XRD patterns of MXenes and Ti_3_AlC_2_. 8](#_Toc166595659)

[**Figure S7.** Optimized structures (top view and side view) of N-TiO_2-x_(B)-MXenes and TiO_2_(B). The Ti, O, C, and N atoms are displayed as the spheres in watchet, red, brown, and dark blue, respectively. 9](#_Toc166595660)

[**Figure S8.** UV-vis adsorption spectra (a) and (b) the optical photographs of Li_2_S_6_ solution treated with different interlayer materials. 9](#_Toc166595661)

[**Figure S9.** (a) SEM images of the conventional Celgard 2400 separator. (b) The bending of N-TiO_2-x_(B)-MXenes modified separator. 10](#_Toc166595662)

[**Figure S10.** (a) Cross-sectional view of the N-TiO_2-x_(B)-MXenes coated separators. (b) Optical pictures of Li_2_S_6_ permeation experiments on different separators. 10](#_Toc166595663)

[**Figure S11.** Optimized structures of different sulfides on the surfaces of N-TiO_2-x_(B)-MXenes and TiO_2-x_(B). The Ti, O, Li, C, N and S atoms are displayed as the spheres in wathet, red, pink, brown, dark blue and yellow, respectively. 11](#_Toc166595664)

[**Figure S12.** CV curves of symmetrical cells with different electrodes at a scan rate of 50 mV s^−1^. 11](#_Toc166595665)

[**Figure S13.** EIS curves of all symmetrical cells. 12](#_Toc166595666)

[**Figure S14.** CV curves of the cells with different interlayers at various scanning rates from 0.1 mV s^−1^ to 0.5 mV s^−1^. 12](#_Toc166595667)

[**F**i**gure S15.** Peak Ι (A), peak Ⅲ (B) and peak Ⅳ (C) in CV curves versus the square root of scan rates. 13](#_Toc166595668)

[**F**i**gure S16.** The TGA in S@CMK-3 cathodes. 13](#_Toc166595668)

[**Figure S17.** Q_L_/Q_H_ ratio of the cell with different interlayers. 13](#_Toc166595669)

[**Figure S18.** GITT voltage profiles of (a) TiO_2-x_(B)-MXenes, (b) TiO_2-x_(B), (c) TiO_2_(B) and (d) Blank cell. 14](#_Toc166595670)

[**Figure S19.** D_Li+_ calculated from the GITT curves of different cathodes. 14](#_Toc166595670)

[**Figure S20.** Discharge/charge curves of the cell at various current rates with different interlayers of (a) TiO_2-X_(B)-MXenes, (b) TiO_2-x_(B), (c) TiO_2_(B) and (d) Blank cell. 15](#_Toc166595671)

[**Figure S21.** EIS curves of all asymmetrical cells before (a) and after cycling (b). 15](#_Toc166595672)

[**Figure S22.** Contact angles between electrolyte and different modified separator. 16](#_Toc166595673)

[**Figure S23.** SEM images of Li anode in bare Li||Li cell (a) and Li||Li cell with N-TiO_2-x_(B)-MXenes interlayer (b) after cycling. 16](#_Toc166595674)

[**Figure S24.** The EIS the of the two Li||Li cells before and after cycling. 16](#_Toc166595675)

[**Table S1.** Battery performances based on different electrocatalysts in this work and recent publications.](#_Toc166595676)  [17](#_Toc166595677)

[**Table S2.** Battery performances at a low current density in comparison of different samples.](#_Toc166595676)  [18](#_Toc166595677)

**Experimental section**

***Preparation of sulfur cathodes***

CMK-3 and S with a weight ratio of 3:7 was added in 20 mL CS_2_ solutions and then sonicated until the CS_2_ solution evaporates completely. The mixture was heated 160 °C in an oven for 24 h under Ar atmosphere to get CMK-3/S. The composites, Super P and PVDF were mixed (with a weight ratio of 8:1:1) in NMP to form a slurry. Then, the slurry was coated on carbon cloth and then dried in an oven at 60 °C for 12 h. The mass loading of S was 2.5 mg cm^−2^~ 7.2 mg cm^−2^.

***Preparation of*** ***different modified separator***

The as-prepared materials and PVDF with a weight ratio of 9:1 was mixed in NMP to form a homogeneous slurry and then coated onto polypropylene (PP) separators (Celgard 2400). The obtained modified separator was dried under vacuum at 60 °C for 12 h and cut into 16 mm circular disks.

***Material characterization***

X-ray diffraction (XRD, Rigaku Ultima IV-185) was used to test the crystal phase of as-prepared interlayer materials with Cu Kα radiation. The morphology and microstructure of these materials were characterized by field emission scanning electron microscopy (FESEM, Quanta 200F) and high angle annular dark field-scanning transmission electron microscopy (HAADF-STEM, JEM-ARM300F, JEOL). The chemical composition of these materials was analyzing by an X‐ ray photoelectron spectroscopy (XPS, PHI QUANTERA-II 11 SXM, and ULVAC-PHI) instrument. The specific surface area, pore volume and pore size distribution of these materials measured through the N_2_ adsorption-desorption data using a 30 Micromeritics ASAP 2020 C to determine at 77 K. UV-vis spectroscopy of the solutions was collected by an ultraviolet and visible spectrophotometer (UV-3600).

***Electrochemical measurements***

CR2032-type coin cells were assembled with as-prepared sulfur cathode, Li anode and different modified separator. The electrolyte was 1 M bis(triffuoromethane) sulfonimide lithium salt (LiTFSI) in a mixed solvent of 1,3-dioxolane (DOL) and 1,2-dimethoxyethane (DME) (1:1 by volume) with 1 wt % LiNO_3_. The E/S ratios ranged from 15.0 μL mg^-1^ to 6.4 μL mg^-1^ for coin cells. The galvanostatic charge-discharge tests at LAND CT2001 battery tester (Wuhan Electronics Co., LTD) with the voltage range of 1.8-2.7 V. Cyclic voltammetry (CV) and the electrochemical impedance spectroscopy (EIS) were investigated on a CHI760E electrochemical workstation (Chenhua instrument Co., China). All specific capacity values and the areal capacity were calculated based on the mass of sulfur. All the electrochemical tests were conducted at 25 ℃.

***Adsorption tests for Li_2_S_6_ solutions***

The Li_2_S and S (molar ratio of 1:5) was added into DME and DOL (1:1 vol) solution under vigorous stirring at 80°C for 24 h to obtain Li_2_S_6_ solution. 30 mg as-prepared materials were added into 6 mL Li_2_S_6_ solution (5.0 mM) and stirred for 10 min followed by room temperature in glove box.

***Symmetric cell assembly***

The electrodes were consisted of as-prepared different materials, Super P, and PVDF with a mass ratio of 8:1:1 in NMP to form uniform slurry. Then the slurry was coated on Al foil and dried in vacuum for 12 h at 60 °C. The as-obtained electrodes were regarded as equivalent working and counter electrodes and 0.2 M Li_2_S_6_ solution (in DME/DOL) containing 1.0 M LiTFSI and 1 wt % LiNO_3_ was used as the electrolyte. Cyclic voltammetry (CV) test was obtained on CHI760E electrochemical workstation between -1.0 V and 1.0 V at scan rates of 0.5 mV s^-1^ and 20 mV s^-1^.

***Li-symmetric cell assembly***

Li-symmetric cell were consisted of with two same electrodes of Li and different modified separator. The electrolyte was 1 M bis(triffuoromethane) sulfonimide lithium salt (LiTFSI) in a mixed solvent of 1,3-dioxolane (DOL) and 1,2-dimethoxyethane (DME) (1:1 by volume) with 1 wt % LiNO_3_.

***Li_2_S nucleation and dissolution measurements***

0.5 M Li_2_S_8_ solution was obtained by dissolving Li_2_S and S (molar ratio of 1:7) in tetraethylene glycol solvent under vigorous stirring at 60°C for 48 h. As-prepared different materials, Super P and PVDF (weight ratio of 8:1:1) were dispersed in NMP and coating on the Al foil with a diameter of 12 mm and dried at 60 °C for 12 h to be used as cathode. 25 μL blank electrolytes were added into the Li side and 25 μL Li_2_S_8_ solutions were added into the cathode side. The cell was discharged galvanostatically to 2.06 V at 0.112 mA, and then discharged potentiostatically at 2.05 V until current decreased to 10^-5^ A. To investigate the dissolution of Li_2_S, fresh cells were first discharged at a current of 0.10 mA to 1.80 V, and subsequently discharged at 0.01 mA to 1.70 V until full conversion of LiPSs into solid Li_2_S. Then, the cells were potentiostatically charged at 2.50 V for the dissolution of Li_2_S into LiPSs until charge current was below 10^-5^ A.

***LSV tests***

LSV was performed in a three-electrode cell. As-prepared different materials coated on glassy carbon as the working electrode, Pt as the counter electrode, Ag/AgCl electrode as the reference, and 0.1 M Li_2_S/methanol solution as the electrolyte. The three-electrode cell was measured at electrochemical workstation at a scan rate of 5 mV s^-1^ between -0.7 and -0.1 V.

***DFT calculations***

We have employed the first-principles^[1a, 1b]^ to perform all density functional theory (DFT) calculations within the generalized gradient approximation (GGA) using the Perdew-Burke-Ernzerhof (PBE)^[2]^ formulation. We have chosen the projected augmented wave (PAW) potentials^[3a, 3b]^ to describe the ionic cores and take valence electrons into account using a plane wave basis set with a kinetic energy cutoff of 500 eV. Partial occupancies of the Kohn−Sham orbitals were allowed using the Gaussian smearing method with a width of 0.1 eV. The electronic energy was considered self-consistent when the energy change was smaller than 10^−6^ eV. A geometry optimization was considered convergent when the energy change was smaller than 0.03 eV Å^−1^. The free energy was calculated using the equation:

G=Eads+ZPE-TS

where G, Eads, ZPE and TS are the free energy, total energy from DFT calculations, zero-point energy and entropic contributions, respectively. hydrogen spillover pathway can be calculated using the nudged elastic band (NEB) method with the transition state of an elementary reaction step. In the NEB method, the path between the reactant(s) and product(s) was discretized into a series structural image. The intermediate images were relaxed until the perpendicular forces were smaller than 0.05 eV/Å.

**Supporting Figures and Tables**

**
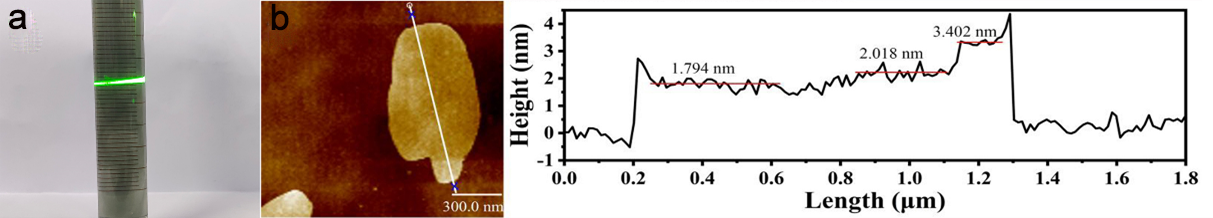
**

Figure S1. (a) The Tyndall effect of the MXenes solution, (b) AFM images of as-prepared few layered Ti_3_C_2_T_x_ MXenes nanosheets.


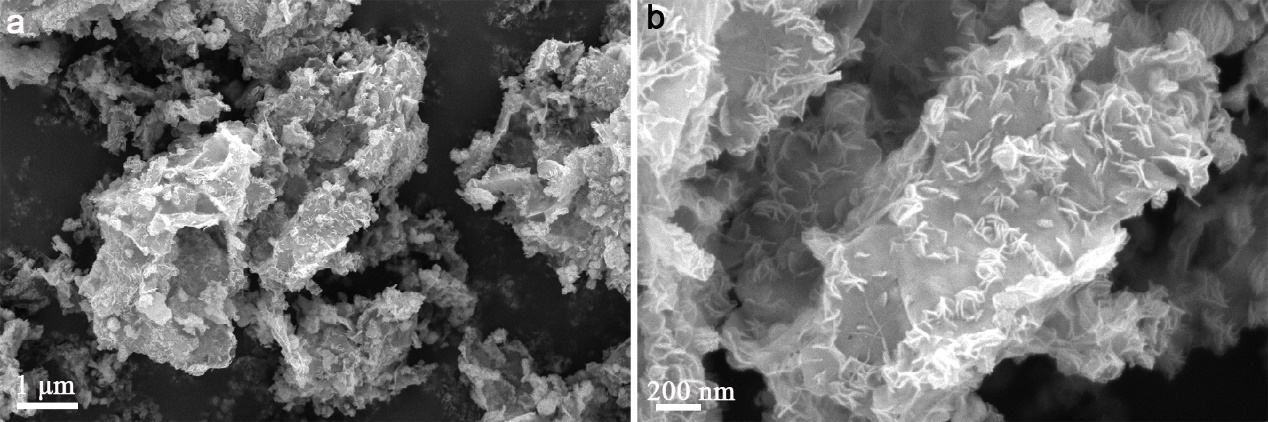


Figure S2. SEM images of TiO_2_(B)-MXenes precursors.


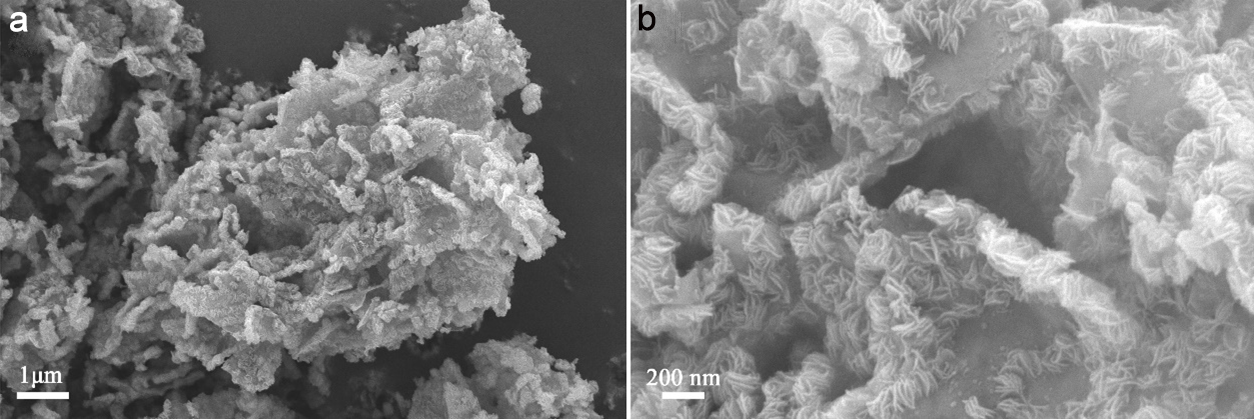


Figure S3. SEM images of as-prepared N-TiO_2-X_(B)-MXenes heterostructures.

**
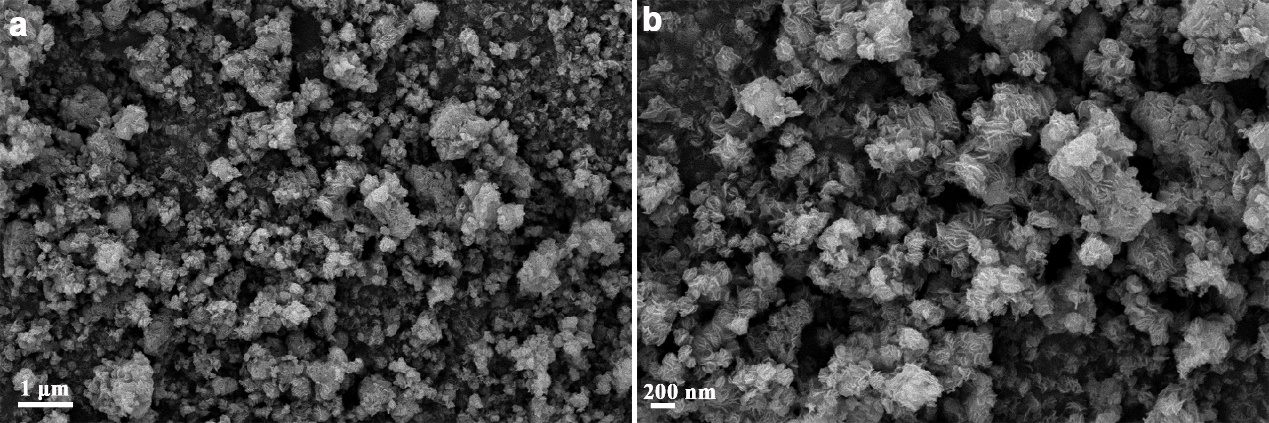
**

Figure S4. SEM images of TiO_2_(B) precursors.


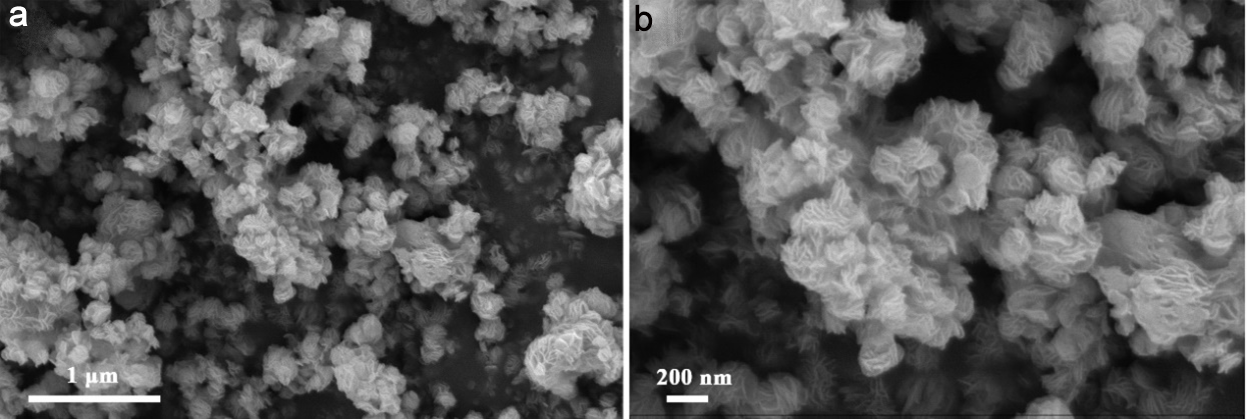


Figure S5. SEM images of TiO_2-x_(B) precursors.


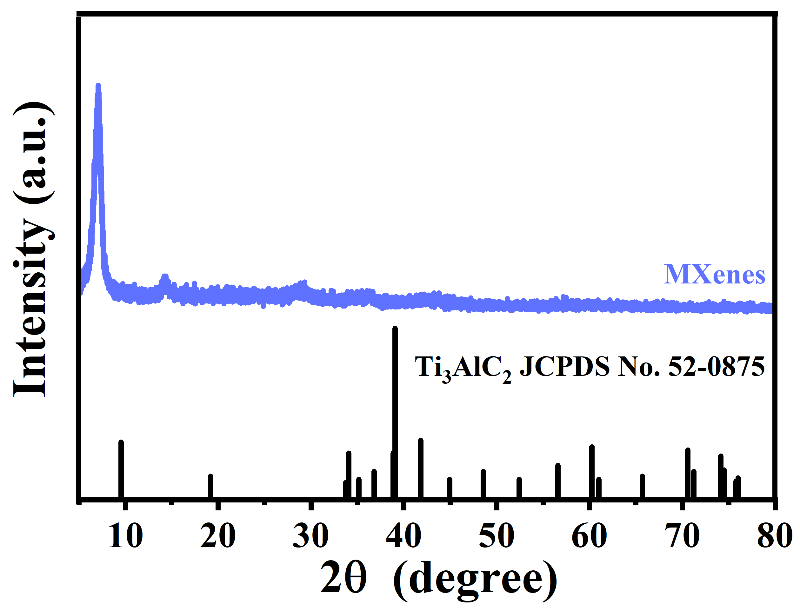


Figure S6. XRD patterns of MXenes and Ti_3_AlC_2_.


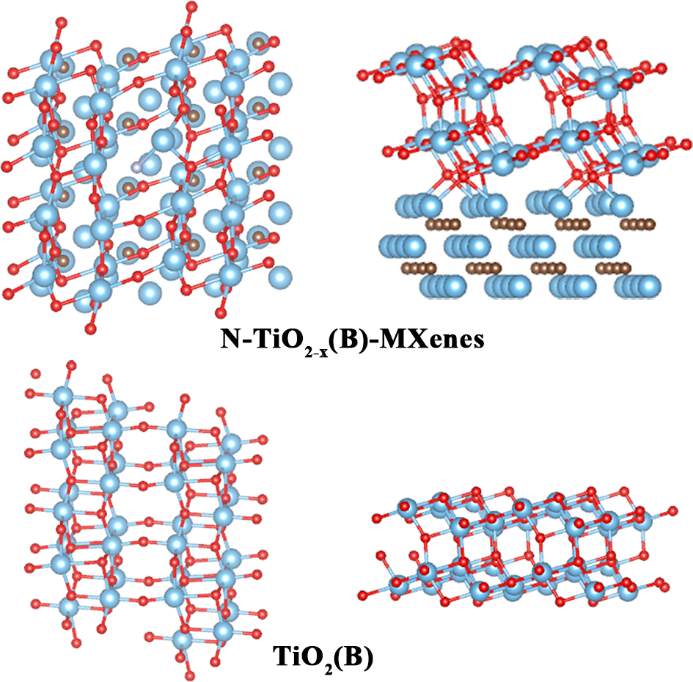


Figure S7. Optimized structures (top view and side view) of N-TiO_2-x_(B)-MXenes and TiO_2_(B). The Ti, O, C, and N atoms are displayed as the spheres in watchet, red, brown, and dark blue, respectively.


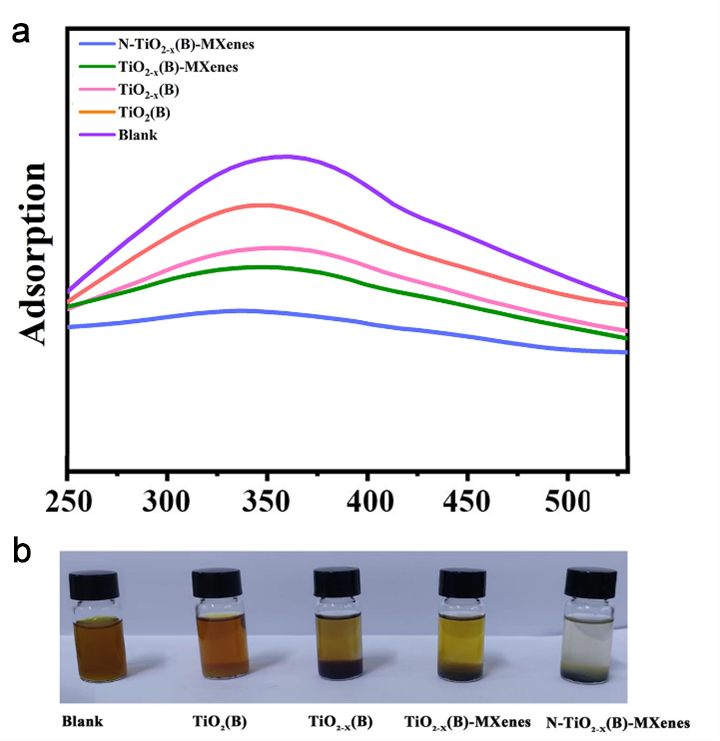


Figure S8. UV-vis adsorption spectra (a) and (b) the optical photographs of Li_2_S_6_ solution treated with different interlayer materials.


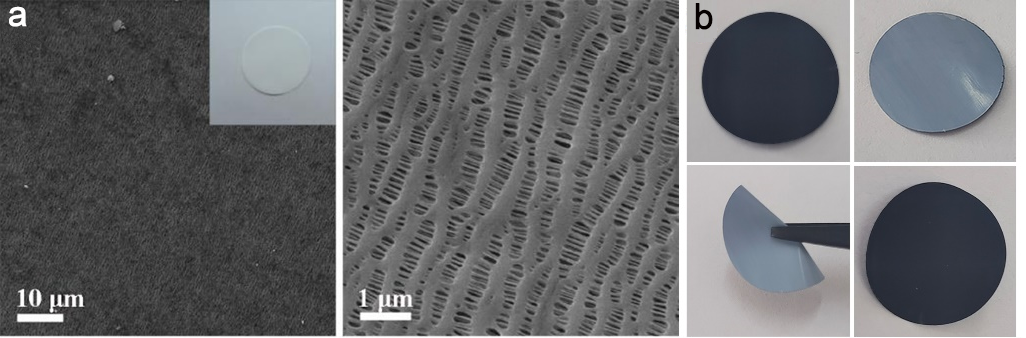
.

Figure S9. (a) SEM images of the conventional Celgard 2400 separator. (b) The bending of N-TiO_2-x_(B)-MXenes modified separator.


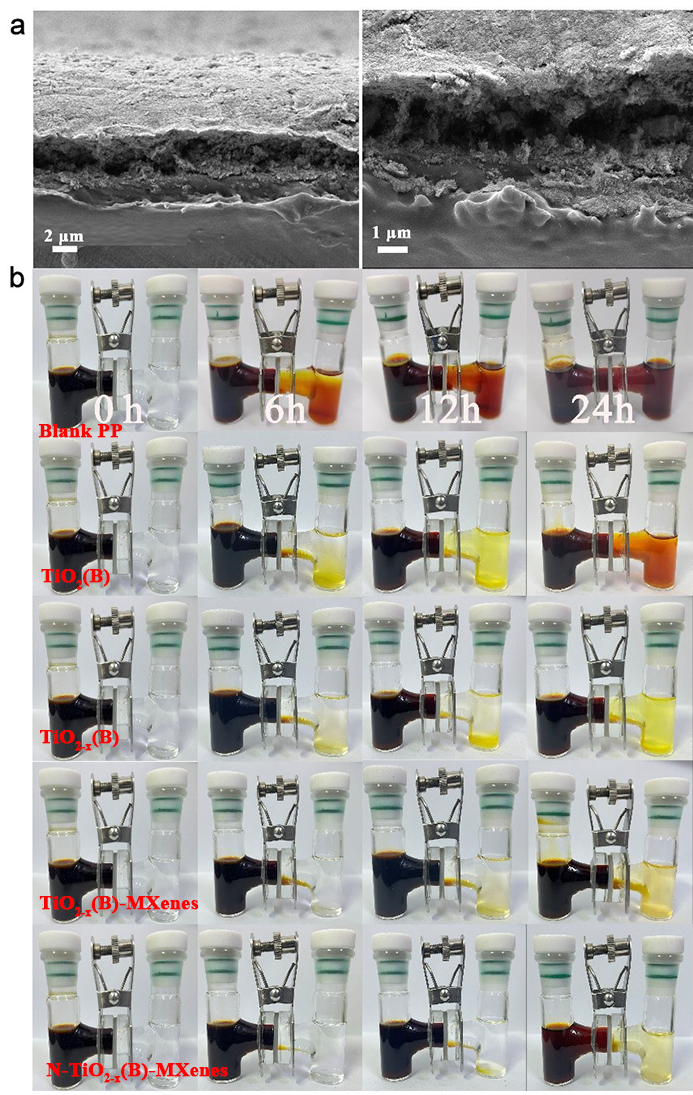


Figure S10. (a) Cross-sectional view of the N-TiO_2-x_(B)-MXenes coated separators. (b) Optical pictures of Li_2_S_6_ permeation experiments on different separators.


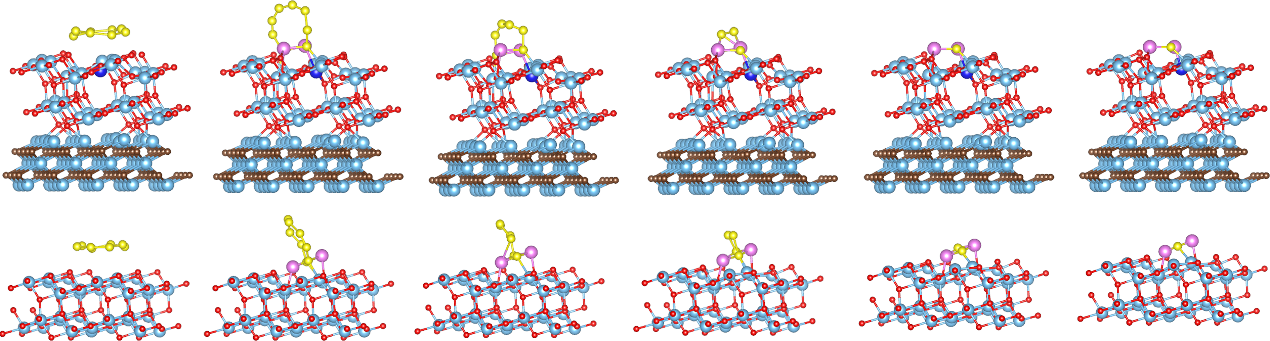
Figure S11. Optimized structures of different sulfides on the surfaces of N-TiO_2-x_(B)-MXenes and TiO_2-x_(B). The Ti, O, Li, C, N and S atoms are displayed as the spheres in wathet, red, pink, brown, dark blue and yellow, respectively.


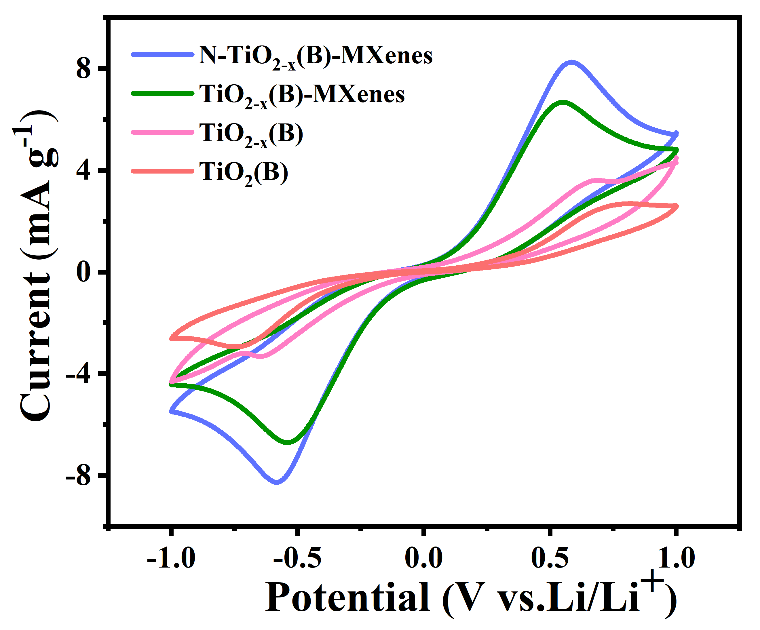


Figure S12. CV curves of symmetrical cells with different electrodes at a scan rate of 50 mV s**^−1^.**

**
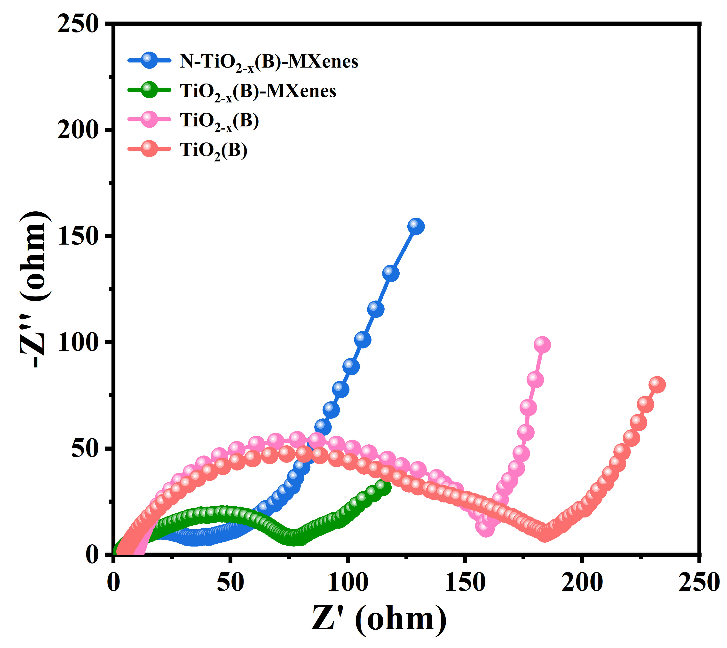
**

Figure S13. EIS curves of all symmetrical cells.


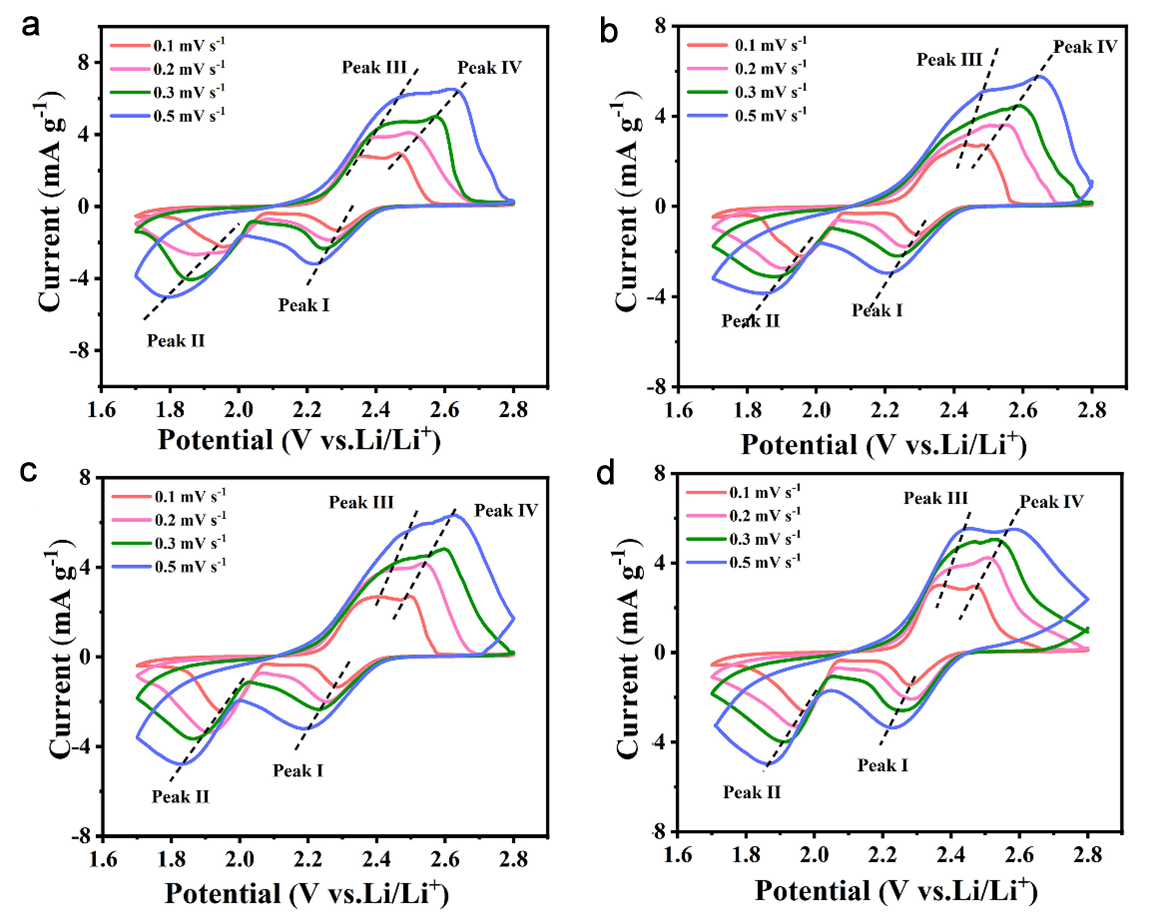


**Figure S14.** CV curves of the cells with different interlayers at various scanning rates from 0.1 mV s^−1^ to 0.5 mV s^−1^.


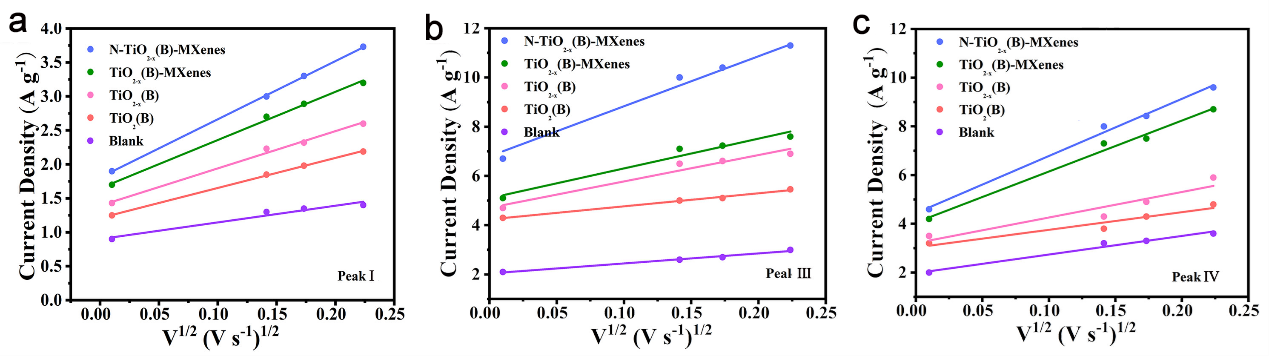
Figure S15. Peak Ι (A), peak Ⅲ (B) and peak Ⅳ (C) in CV curves versus the square root of scan rates.


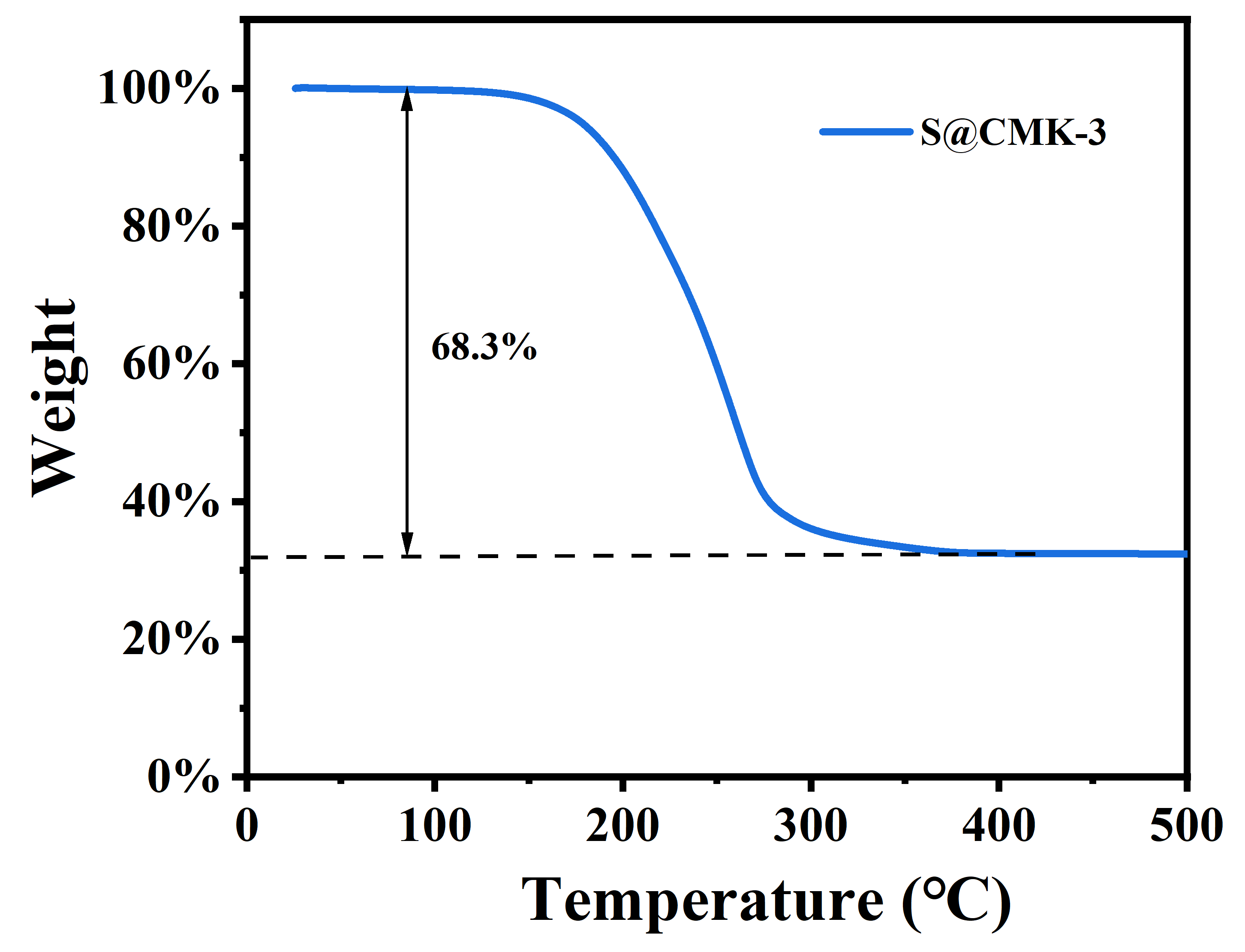


**Figure S16.** The TGA curves in S@CMK-3 cathodes.


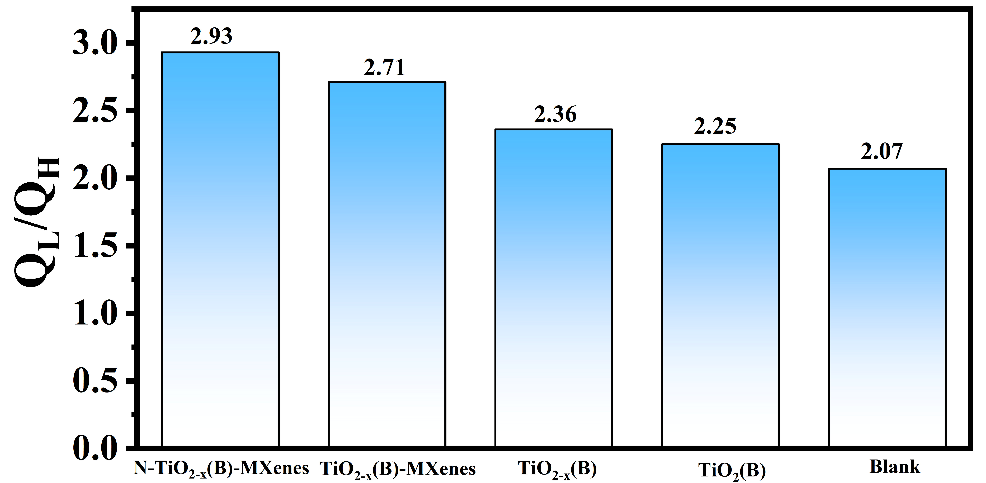


Figure S17. Q_L_/Q_H_ ratio of the cell with different interlayers.

**
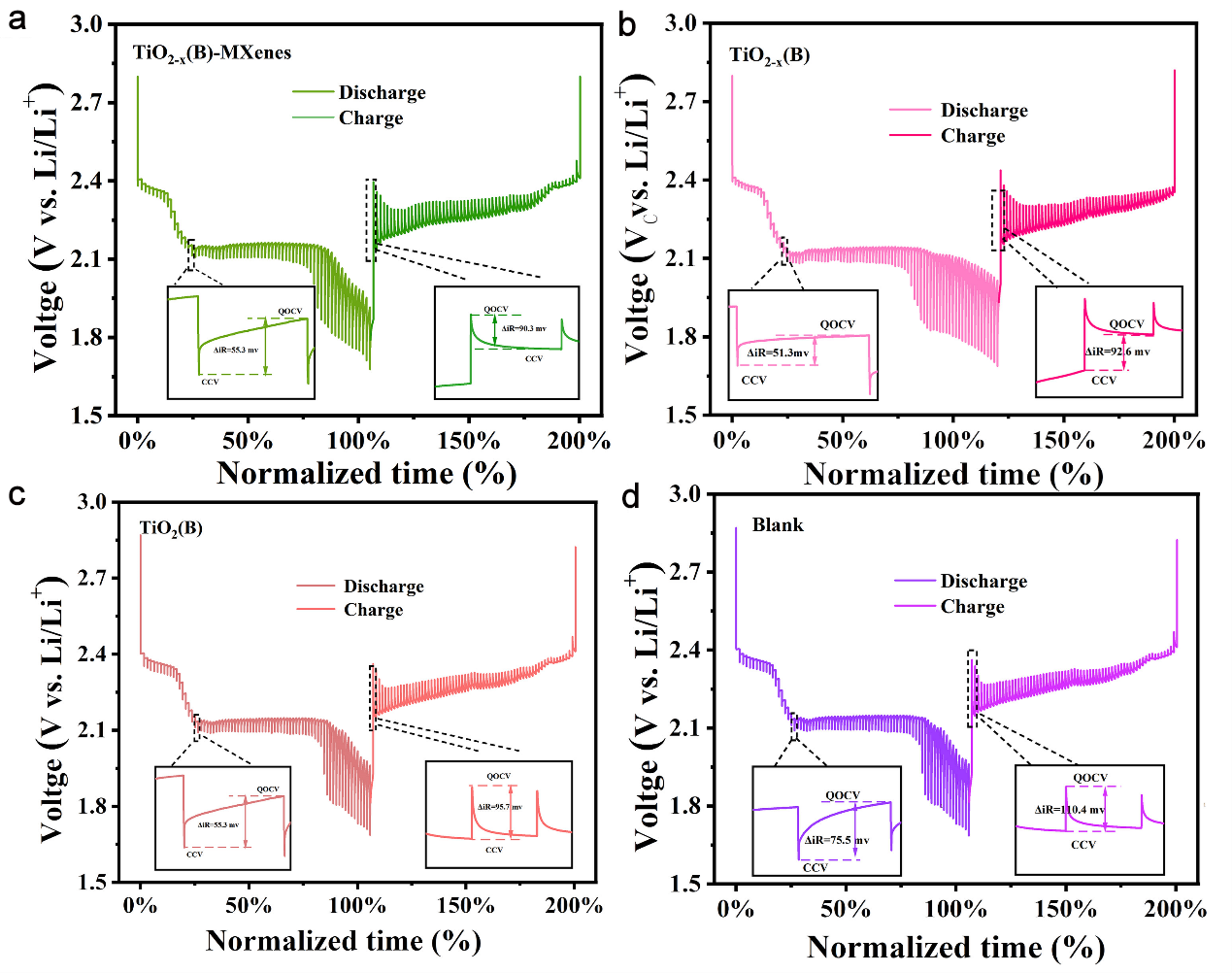
**Figure S18. GITT voltage profiles of (a) TiO_2-x_(B)-MXenes, (b) TiO_2-x_(B), (c) TiO_2_(B) and (d) Blank cell.


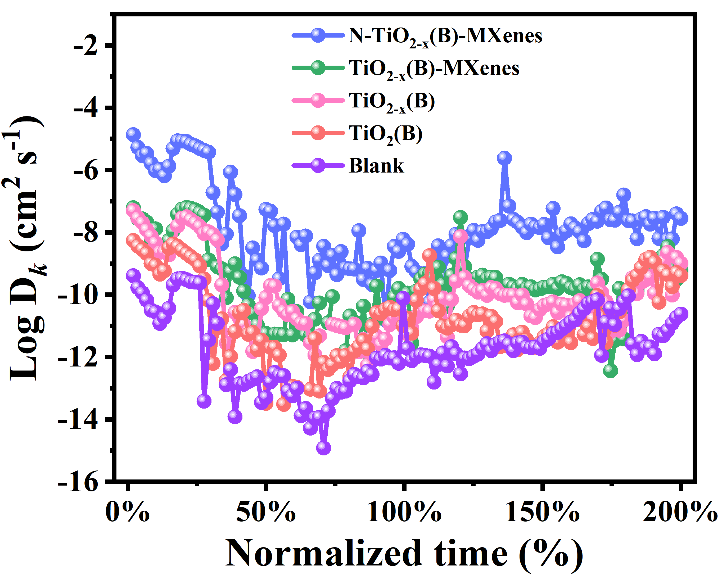


Figure S19. D_Li+_ calculated from the GITT curves of different cells.

**
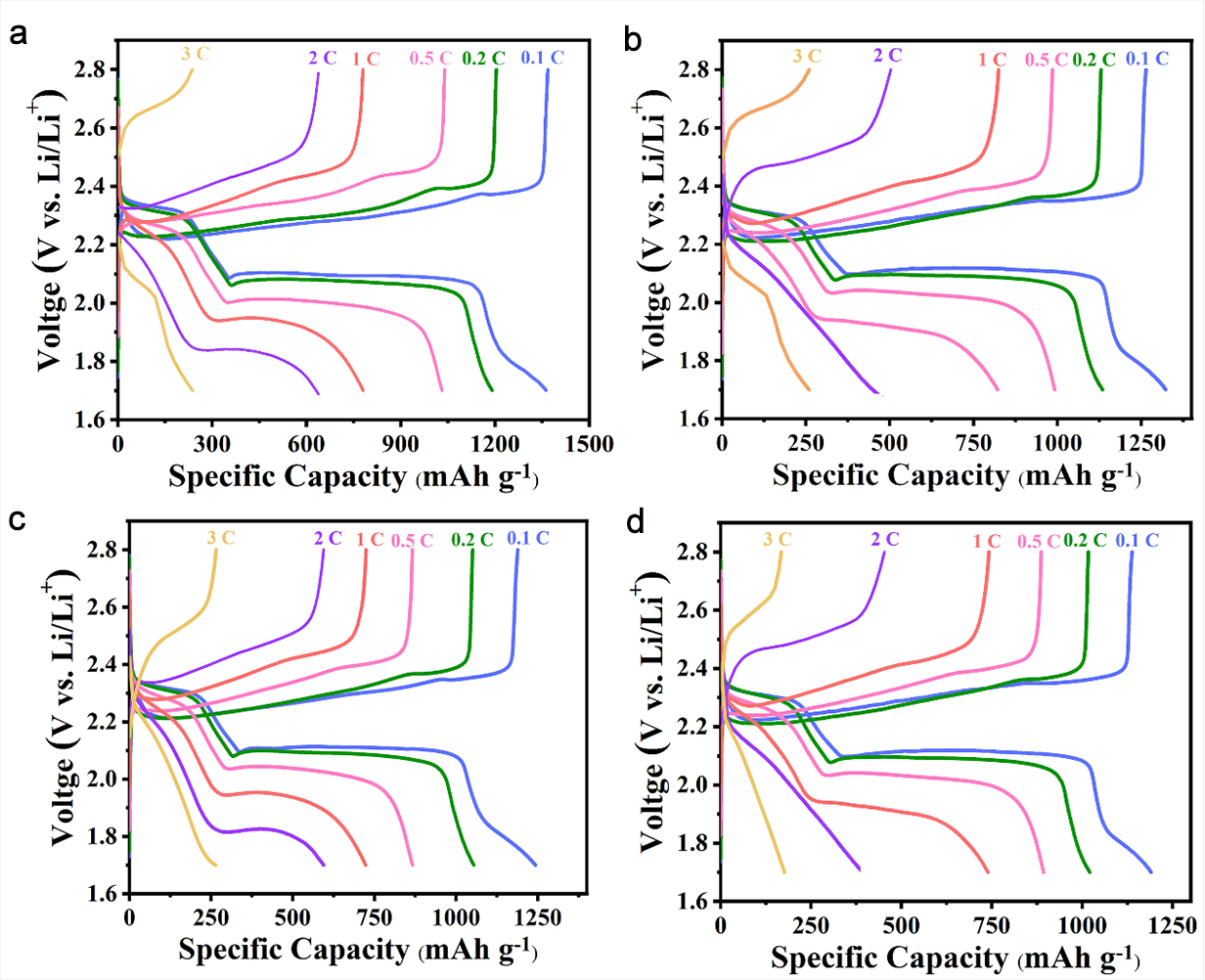
**

Figure S20. Discharge/charge curves of the cell at various current rates with different interlayers of (a) TiO_2-X_(B)-MXenes, (b) TiO_2-x_(B), (c) TiO_2_(B) and (d) Blank cell.


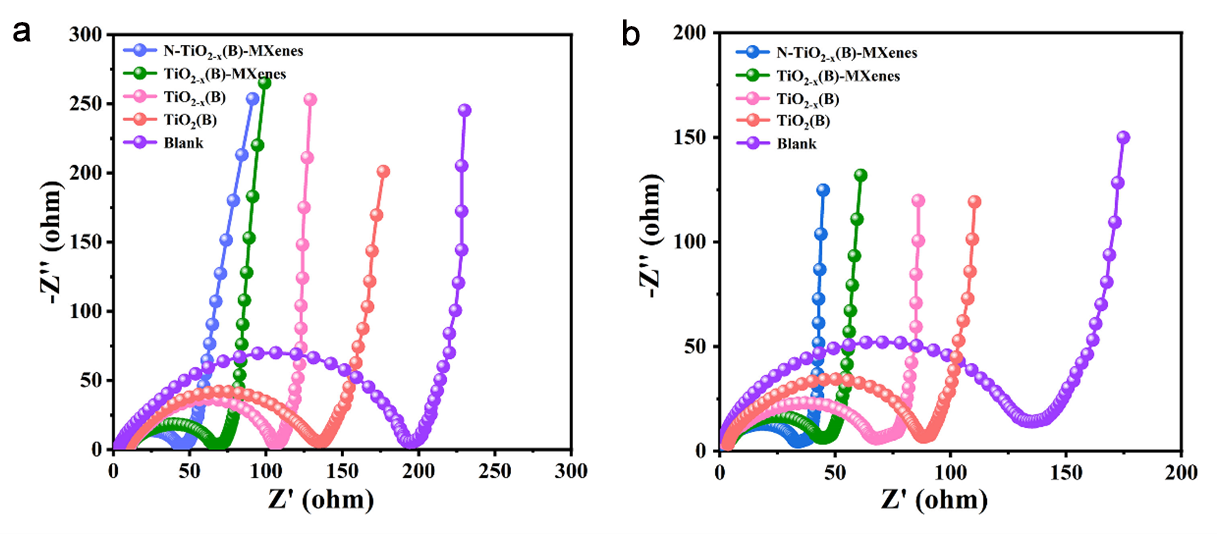


Figure S21. EIS curves of all asymmetrical cells before (a) and after cycling (b)**.**


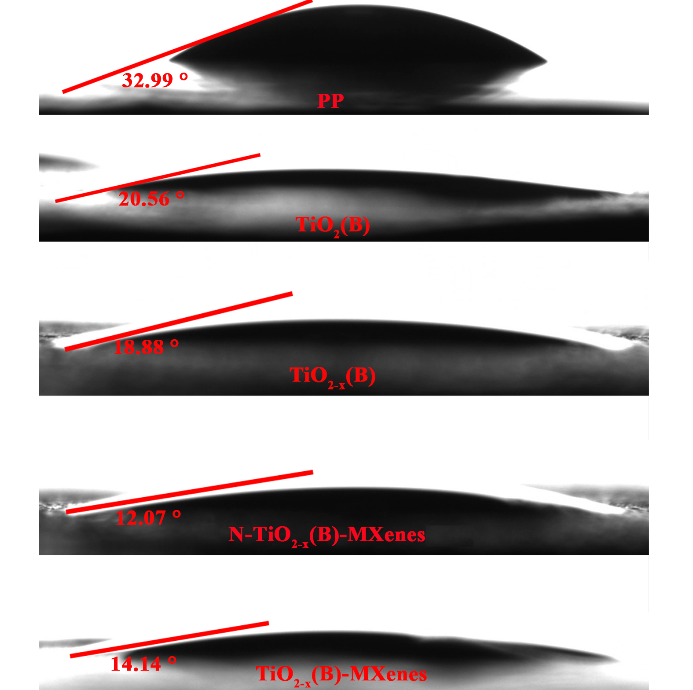


Figure S22. Contact angles between electrolyte and different modified separator.


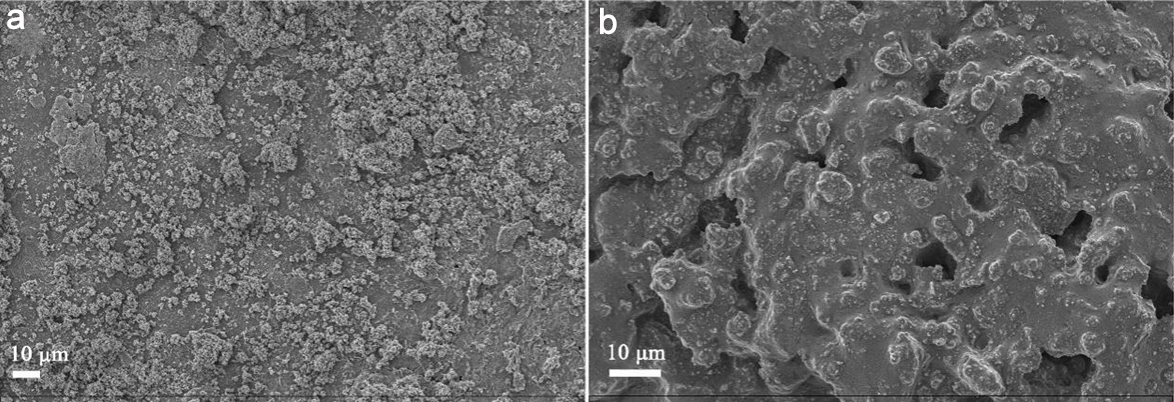


Figure S23. SEM images of Li anode in bare Li||Li cell (a) and Li||Li cell with N-TiO_2-x_(B)-MXenes interlayer (b) after cycling.

**
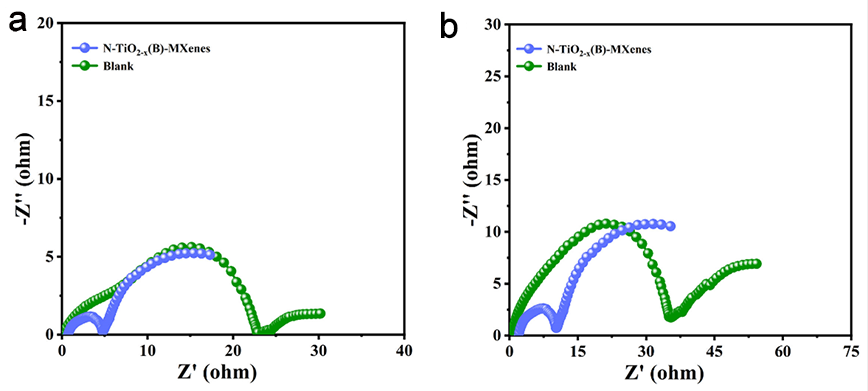
**

Figure S24. The EIS the of the two Li||Li cells before and after cycling.

Table S1. Battery performances based on different electrocatalysts in this work and recent publications.

| Material | Sulfur loading  (mg cm^−2^) | E/S ratio  (μL mg^−1^) | Areal capacity  (mAh cm^−2^) | Current/Cycle  number | Ref. |
| --- | --- | --- | --- | --- | --- |
| N-TiO_2-X_(B)-MXenes | 2.5 | 15 | 1.67 | 1C/600 | This work |
|  | 2.5 | 15 | 3.02 | 0.1C/60 |  |
|  | 7.2 | 6.4 | 6.15 | 0.1C/50 |  |
| CNT@TiO_2-x_ | 2.2 | 12 | 1.30 | 1C/500 | [4] |
| TiO | 2.0 | -- | 1.50 | 0.3C/50 | [5] |
| TiO_2_-TiN | 3 | -- | 1.90 | 0.3C/100 | [6] |
| TiO_2_–Ni_3_S_2_/rGO | 1.92 | 20 | 1.22 | 0.5C/900 | [7] |
| TiO_2-_*_x_*@Ni | 1.5 | -- | 1.20 | 1C/500 | [8] |
| OV–T*_n_*QDs@PCN | 2.2 | 17 | 1.54 | 0.1C/100 | [9] |
| SA/N–Ti_3_C_2_T*_x_* | 5.02 | 15 | 3.71 | 0.1/50 | [10] |
| V_2_C MXene | 4.8 | 7 | 3.84 | 0.1C/50 | [11] |
| A/R-TiO_2_ | 5.0 | -- | 5.46 | 0.1C/80 | [12] |
| TiO_2-x_@Ni | 1.5 | -- | 1.20 | 1C/500 | [13] |
| TiO | 3.5 | -- | 2.87 | 0.5/500 | [14] |
| CNT@TiO_2−x_ | 2.2 | 12 | 5.4 | 1C/300 | [15] |
| D-TiO_2_ | 3.2 | -- | 3.36 | 1C/100 | [16] |

Tables S2. Battery performances at a low current density in comparison with different samples.

| Material | Cycle Number | Discharge capacity  (mAh g^-1^) | Capacity Retention | Ref. |
| --- | --- | --- | --- | --- |
| N-TiO_2-x_(B)-MXenes | 60 | 1216.7 | 90.0% | This work |
| Ni-CeO_2_-CNF | 100 | 970 | 82.3% | [6] |
| Co_3_O_4_/ZnO | 100 | 1023 | 78.6% | [17] |
| OMC-g-MXene | 50 | 1074.5 | 87.2% | [18] |
| TiN@CNFs | 100 | 1110 | 79.9% | [19] |
| NiMoO_4_@NSCC | 80 | 995.6 | 86.3% | [20] |
| S/Fe_3-x_C | 50 | 1089 | 85.6% | [21] |
| Fe-N_5_-C | 100 | 924.1 | 75.2% | [22] |
| NiCo-LDH@rGO | 100 | 999.3 | 74.8% | [23] |
| Mo_2_C−MoO_3_ | 50 | 1160 | 81.6% | [24] |
| Fe-NSC | 100 | 1016 | 80.7% | [25] |

**References**

[1] a) G. Kresse, J. Furthmüller, *Comput. Mater. Sci.* **1996**, *6*, 15; b) Kresse, Furthmuller, *Phys. Rev. B* **1996**, *54*, 11169.

[2] n. Perdew, n. Burke, n. Ernzerhof, *Phys. Rev. Lett.* **1996**, *77*, 3865.

[3] a) E. Torres, T. P. Kaloni, *Comput. Mater. Sci.* **2020**, *171*, 109237; b) H. Levämäki, M. Kuisma, K. Kokko, *J. Chem. Phys.* **2019**, *150*, 054101.

[4] Y. Wang, R. Zhang, J. Chen, H. Wu, S. Lu, K. Wang, H. Li, C. J. Harris, K. Xi, R. V. Kumar, S. Ding, *Adv. Energy Mater.* **2019**, *9*, 1900953.

[5] M. Cui, Z. Zheng, J. Wang, Y. Wang, X. Zhao, R. Ma, J. Liu, *Energy Storage Mater.* **2021**, *35*, 577.

[6] P. Xue, K. Zhu, W. Gong, J. Pu, X. Li, C. Guo, L. Wu, R. Wang, H. Li, J. Sun, G. Hong, Q. Zhang, Y. Yao, *Adv. Energy Mater.* **2022**, *12*, 2200308.

[7] R. Wang, C. Luo, T. Wang, G. Zhou, Y. Deng, Y. He, Q. Zhang, F. Kang, W. Lv, Q.-H. Yang, *Adv. Mater.* **2020**, *32*, 2000315.

[8] P. Zhang, Y. Zhao, Y. Li, N. Li, S. R. P. Silva, G. Shao, P. Zhang, *Adv. Sci.* **2023**, *32*, 2000315.

[9] H. Zhang, L. Yang, P. Zhang, C. Lu, D. Sha, B. Yan, W. He, M. Zhou, W. Zhang, L. Pan, Z. Sun, *Adv. Mater.* **2021**, *33*, 2008447.

[10] H. Gu, W. Yue, J. Hu, X. Niu, H. Tang, F. Qin, Y. Li, Q. Yan, X. Liu, W. Xu, Z. Sun, Q. Liu, W. Yan, L. Zheng, Y. Wang, H. Wang, X. Li, L. Zhang, G. Xia, W. Chen, *Adv. Energy Mater.* **2023**, *13*, 2204014.

[11] L. Chen, Y. Sun, X. Wei, L. Song, G. Tao, X. Cao, D. Wang, G. Zhou, Y. Song, *Adv. Mater.* **2023**, *35*, 2300771.

[12] L. Ma, Y. Zhang, S. Zhang, L. Wang, C. Zhang, Y. Chen, Q. Wu, L. Chen, L. Zhou, W. Wei, *Adv. Funct. Mater.* **2023**, *33*, 2305788.

[13] P. Zhang, Y. Zhao, Y. Li, N. Li, S. R. P. Silva, G. Shao, P. Zhang, *Adv. Sci.* **2023**, *10*, 2206786.

[14] N. Li, L. Yu, J. Xi, *Small* **2021**, *17*, 2103001.

[15] Y. Wang, R. Zhang, J. Chen, H. Wu, S. Lu, K. Wang, H. Li, C. J. Harris, K. Xi, R. V. Kumar, S. Ding, *Adv. Energy. Mater.* **2019**, *9*, 1900953.

[16] Q. He, B. Yu, H. Wang, M. Rana, X. Liao, Y. Zhao, *Nano Research* **2020**, *13*, 2299.

[17] B. Wang, Y. Ren, Y. Zhu, S. Chen, S. Chang, X. Zhoua, P. Wanga, H. Sun, X. Menga, S. Tanga, *Adv. Sci.* **2023**, *10*, 2300860.

[18] X. Li, Q. Guan, Z. Zhuang, Y. Zhang, Y. Lin, J. Wang, C. Shen, H. Lin, Y. Wang, L. Zhan, L. Ling, *ACS Nano* **2023**, *17*, 1653.

[19] Y. Yao, H. Wang, H. Yang, S. Zeng, R. Xu, F. Liu, P. Shi, Y. Feng, K. Wang, W. Yang, X. Wu, W. Luo, Y. Yu, *Adv. Mater.* **2019**, *32*, 190565.

[20] T. Sun, X. Zhao, B. Li, H. Shu, L. Luo, W. Xia, M. Chen, P. Zeng, X. Yang, P. Gao, Y. Pei, X. Wang, *Adv. Funct. Mater.* **2021**, *31*, 2101285.

[21] Y. Zhang, G. Li, J. Wang, G. Cui, X. Wei, L. Shui, K. Kempa, G. Zhou, X. Wang, Z. Chen, *Adv. Funct. Mater.* **2020**, *30*, 2001165.

[22] Y. Zhang, J. Liu, J. Wang, Y. Zhao, D. Luo, A. Yu, X. Wang, Z. Chen, *Angew. Chem. Int. Ed.* **2021**, *60*, 26622.

[23] W. Qiu, G. Li, D. Luo, Y. Zhang, Y. Zhao, G. Zhou, L. Shui, X. Wang, Z. Chen, *Adv. Sci.* **2021**, *8*, 2003400.

[24] Q. Bin, C. Yifei, W. Pengcheng, Z. YongChun, C. Jian, Q. Junlei, *Energy Storage Mater.* **2022**, *47*, 345.

[25] L. Ren, J. Liu, Y. Zhao, Y. Wang, X. Lu, M. Zhou, G. Zhang, W. Liu, H. Xu, X. Sun, *Adv. Funct. Mater.* **2023**, *33*, 2210509.
